# The complexity of understanding others as the evolutionary origin of empathy and emotional contagion

**DOI:** 10.1038/s41598-019-41835-5

**Published:** 2019-04-08

**Authors:** Fabrizio Mafessoni, Michael Lachmann

**Affiliations:** 1 Max Planck for Evolutionary Anthropology, Department of Evolutionary Genetics, Leipzig, 04103 Germany; 2 Santa Fe Institute, Santa Fe, New Mexico 87501 USA

## Abstract

Contagious yawning, emotional contagion and empathy are characterized by the activation of similar neurophysiological states or responses in an observed individual and an observer. For example, it is hard to keep one’s mouth closed when imagining someone yawning, or not feeling distressed while observing other individuals perceiving pain. The evolutionary origin of these widespread phenomena is unclear, since a direct benefit is not always apparent. We explore a game theoretical model for the evolution of mind-reading strategies, used to predict and respond to others’ behavior. In particular we explore the evolutionary scenarios favoring simulative strategies, which recruit overlapping neural circuits when performing as well as when observing a specific behavior. We show that these mechanisms are advantageous in complex environments, by allowing an observer to use information about its own behavior to interpret that of others. However, without inhibition of the recruited neural circuits, the observer would perform the corresponding downstream action, rather than produce the appropriate social response. We identify evolutionary trade-offs that could hinder this inhibition, leading to emotional contagion as a by-product of mind-reading. The interaction of this model with kinship is complex. We show that empathy likely evolved in a scenario where kin- and other indirect benefits co-opt strategies originally evolved for mind-reading, and that this model explains observed patterns of emotional contagion with kin or group members.

## Introduction

Learning enables organisms to adapt flexibly to their environment without waiting for natural selection to take its long and arduous route. However, the more complex the environment, the slower the adaptive gain from either learning or evolution. One of the most complex and relevant environments that organisms encounter is the social milieu. Here individuals are presented with a multitude of other individuals, each with a complex adaptive responses. Predicting these “black boxes”, a process called mind-reading^1^, is hard. Thus accurate social predictions would require a long adaptive process, requiring accurate information about each perceived stimulus and the possible responses. Luckily a shortcut is available: the organism holds an almost identical copy of the “black box” - its own decision making apparatus or neural circuitry, which can be used to extrapolate inferences concerning others’ behavior. Information acquired over evolutionary time and information acquired through individual learning can thus be used to speed up adaptation to social encounters^2^.

Since the discovery of mirror neurons^3,4^, a large number of neurophysiological studies have shown that similar brain regions are activated when observing and when performing a specific action, or perceiving a given emotional or sensory stimulus^4–8^. These mirroring phenomena supported the view that an *observer* can decipher an *actor*’s actions and states through at least a partial simulation of those same neural circuits and internal states elicited as an actor, in what de Waal termed “perception-action mechanisms” PAMs^9,10^. Neuroscientists proposed that these brain regions, activated both *as observers* or *as actors*, possibly underlie “shared representations” of the perceived stimuli and actions^11^. For an observer, a potential advantage of activating the as-actor neural configuration is to have access to information about one’s own sensory and motor programs. In the social context this information might help to interpret social cues and infer an observed actor’s actions or intentions^12^, allowing to solve the complex “black box” problem. We adopt here the term “simulation” to indicate any mind-reading strategy that relies on self-information experienced as an actor, rather than information acquired via observation of others^10^.

**Figure 1:**
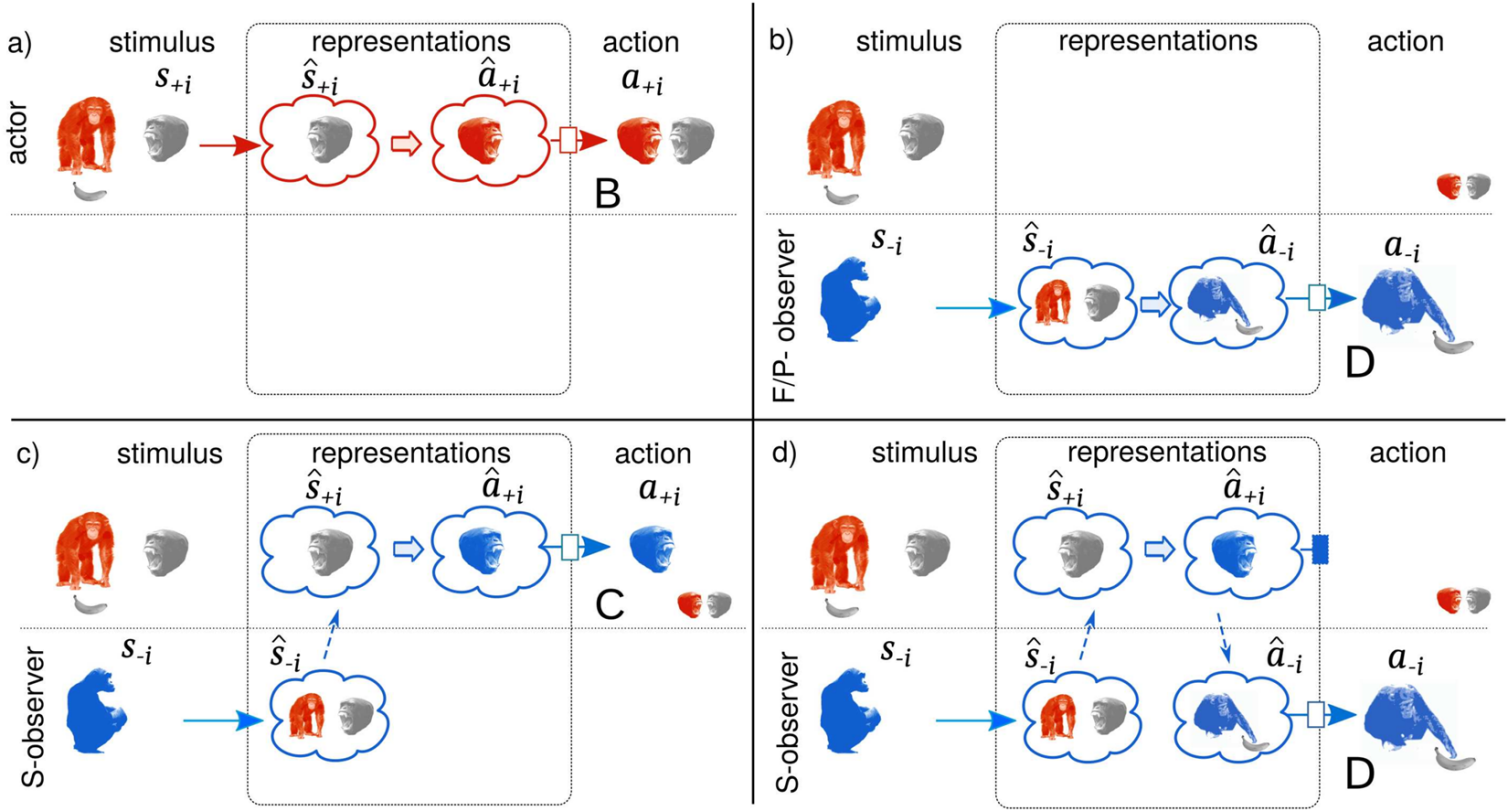
Cognitive circuits connecting stimuli (*s* ∈ *S*), actions (*a* ∈ *A*) and their representations ($\hat{s}\in \hat{S}$ and *â* ∈ *Â*), for a contagious distress example. An actor (in red) perceives a dangerous stimulus (*s*_+*i*_ e.g. a social interaction with an aggressive individual, in gray) and associates (thick arrow) its representation ${\hat{s}}_{+i}$ to a neural circuit underlying a fight-or-flight response ${\hat{a}}_{+i}$, in turn responding aggressively and defending itself (action *a*_+*i*_, *B*). (**b**–**d**) An observer (in blue) perceives (**a**) as a social stimulus *s*_−*i*_ and can perform an appropriate response *D* (avoiding the conflict and possibly exploiting it to steal/forage resources) through different strategies: *F*, *P* and *S*. (**b**) *F* and *P*-strategies: by observing many similar social stimuli – over a lifetime or over evolutionary time– the observer learns (*P*) or evolves (*F*) to map (thick arrow) the social stimulus to the representation of the optimal action *a*_−*i*_. (**c,d**) *S*-strategy: the as-actor representations are recruited to infer an actor’s response ${\hat{a}}_{+i}$, via the simulation functions (dashed arrows, *γ*_*s*_ upward, *γ*_*a*_ downward). (**c**) When ${\hat{a}}_{+i}$ is uninhibited, the observer responds with ${a}_{+i}$ (*C*, contagious distress) with potential costs for itself (fight, energy) and benefits for the actor (help in the conflict). (**d**) When inhibited, â_+*i*_ is mapped to a representation of the optimal response for the observer ${\hat{a}}_{-i}$, and this response is executed (*D*). In all figures arrows indicate cognitive functions as described in Table 2: stimulus-action mappings (learning functions) as thick arrows, activation functions as barred arrows (dark with inhibition, empty with activation), simulation functions as dashed arrows.

Simulative strategies face possible disadvantages and computational obstacles: the secondary activation of as-actor neural circuits during observation has to be discriminated precisely from primary activations of the same circuits when performing the corresponding action oneself. Otherwise, the partial activation of neural configuration which are usually used in the as-actor context may prime the corresponding autonomic and somatic responses, and unless properly inhibited would then evoke in the observer the responses of an actor^4,9,13^. We define these events in which a simulative observer performs the same action as an observed actor as accidental coordination (*C*; Fig. 1a–d). A pathological lack of inhibition results in compulsive imitation, as in echopraxia and echolalia, the involuntary repetition of others’ actions or language^14^, respectively. In specific situations, accidental coordination can be advantageous. However in the context of mind-reading and social interactions, individuals are often characterized by different states or competing motivations, thus for an observer it is generally not advantageous to copy blindly the action of an observed actor. The potential cost of accidental coordination is two-folded: first, it hinders the observer from performing the most appropriate response to an actor’s behavior; second, it might imply further specific costs: for instance, contagious distress^15,16^ can interfere with an optimal decision making, and potentially lead to costly actions, such as alarm calls or aggressive behavior (Fig. 1c). A well documented example, in which the first inherent cost is apparent, is *motor interference*, in which the observation of a movement impairs the performance of an incongruent movement in the observer^13^. For this reason, the inhibition of accidental coordination is likely shaped by natural selection, and of primary importance in mind-reading and mirroring processes^14,17^.

Previous theoretical models of the evolution of emotional contagion restricted their attention to cases in which coordination is advantageous^18,19^. Ackay *et al*. showed that other-regarding preferences are evolutionarily stable only when payoffs are synergistic between two individuals^18^. Nakahashi and Ohtsuki proposed a model in which emotional contagion works as a social learning strategy: for an observer it is advantageous to copy the emotion of an observed actor when it needs to respond analogously to a stimulus, but noise or differences between the two individuals hinder an exact copy of the observed behavior^19^.

Here we relax this assumption, allowing for the presence of costs in accidental coordination, modeling the antagonistic interactions between actor and observer and the divergence of their motivations as dis-coordination games. Following a game-theoretical approach similar to Nakahashi and Ohtuski^19^ we contrast several mind-reading strategies and show that in complex social contexts, where there may be insufficient social information to infer others’ behavior, simulative strategies will evolve to improve the ability to infer others’ actions. A by-product of this simulation is accidental coordination, possibly in the form of emotional contagion, even when this coordination is costly.

In the second part of the paper, we investigate the effects of assortment mechanisms such as kin and the implication for what regards the evolution of empathy. We emphasize that we do not aim at explaining all forms of prosocial behavior: here we focus on a strict definition of empathy, i.e. prosocial behavior that is mediated by the activation of similar neurophysiological responses in the cooperator and in the recipient individual^9,20,21^ and we do not make claims in regards to other forms of prosocial behavior, such as sympathy, i.e. prosocial attitudes not mediated by the as-actor network.

For clarity, we presented the model in terms of specific cognitive functions. However, note that the trade-offs that we identify are not specific to this implementation. This is shown with an evolutionary invasion analysis presented in Appendix section 2, and by implementing different variations of the model (Appendix section 2.4, section 4). In the discussion we will return to the question of the model’s generality.

## Model

### Interactions

We adopt an evolutionary game theory approach to investigate the evolution of a population of individuals genetically adopting different mind-reading strategies, i.e. strategies adopted by an observer to react to an observed actor. We first describe under which circumstances simulative strategies, denoted by *S*, would evolve against competing strategies, either relying on fixed evolved responses (F-strategies) or on learning (P-strategies). Later, we perform an evolutionary invasion analysis, examining how traits determining how simulative strategies inhibit coordination would evolve. We validate these results also with agent-based simulations (Appendix, section 6). We explore a simple evolutionary setup with pairwise interactions: each individual in the population will, at times, be an actor (Fig. 1a), who simply responds to an environmental stimulus, and at other times an observer (Fig. 1b–d), who responds to an actor on the base of its own mind-reading strategy. Hence, at each step, each individual is either an unpaired actor, with probability 1 − 2*p*, or is paired, with probability 2*p*, in which case it has 50% chance to be the actor, and 50% chance to be the observer. An observer is here defined as an individual potentially able to respond to an actor’s behavior, thus high values of *p* indicate accurate social cues as well as a high proportion of social interactions.

Both actors and observers perceive a stimulus *s* ∈ *S*, and respond with an action *a* ∈ *A*. We denote as-actor and as-observer elements with positive and negative subscripts, respectively. Thus an actor *y*^+^ receives a stimulus *s* = *s*_+*i*_ ∈ *S*^+^, whereas an observer *y*^−^ perceives the corresponding social cue/stimulus *s* = *s*_−*i*_ ∈ *S*^−^. A social cue *s*_−*i*_ can be interpreted either as the general perception of the context “actor + stimulus *s* + *i* for the actor”, or as the perception of a social cue suggesting the internal state of the actor.

### Fitness

For each stimulus, we denote the best action for the focal individual, providing the largest payoff, with the same index. Hence, for an actor perceiving stimulus *s*_+*i*_, the best action is *a*_+*i*_, which we also denote as response *B* and provides a payoff *b*; for an observer with stimulus *s*_*−i*_ the best action is *a*_*−i*_, denoted as *D*, and provides a payoffs *d*^−^ (Table 1). Other actions with *i* ≠ *j*, here defined as either ∅ or 0 for actors and observers respectively, provide lower payoffs, here considered for simplicity as zero. A special case occurs when an observer performs the same action *a*_+ *i*_ as the actor, an event that is denoted as C, for coordination. This can occur when the observer activates the same neural circuit as the actor and does not inhibit it (see below *Mind-reading strategies*).

**Table 1:** Payoff matrix for the responses of an actor and an observer to a stimulus *i*.

actor	*B*(*a* = *a*_+*i*_)	${\boldsymbol{\varnothing }}$(*a* = *a*_*j*_ _≠ +*i*_)
observer		
*D*(*a* = *a*_−*i*_)	*b* − *d*^+^	0
	*d* ^−^	0
0(*a* = *a*_*j*__≠__±*i*_)	*b*	0
	0	0
*C*(*a* = *a*_+*i*_)	*b* + *c*^+^	0
	−*c*^*−*^	0

To be conservative against invasion by the simulative strategy, we assumed a Prisoner’s Dilemma-like payoff structure: optimal observer responses, *D* for defection, provide the highest payoffs to the observer while coordination, *C*, is always worse than *D* and 0. For the actor, observer action *C* gives the highest payoff, and *D* the lowest. This matrix allows us to represent both cheap coordination, when *c*^+^ ≈ *c*^−^ ≈ 0, and more costly coordination, when *c*^−^ > 0. We note that the latter, being potentially beneficial for the actor and costly for the observer, is structurally similar to cooperation, hence we retain a similar cooperation-defection notation. Low cost coordination (*c*^−^ ≈ *c*^+^ ≈ 0) can represent phenomena such as emotional contagion and contagious yawning; more costly coordination (*c*^−^ > 0) and recipient’s benefits (*c*  > 0) can reflect forms of empathy-driven cooperation.

We consider an evolutionary-dynamics setting with additive payoffs, where the absolute fitness of a focal phenotype is simply given by the average probability of interactions resulting in *B*, ∅, *C*, *D* and 0 events, and their payoffs. We define the expectation of these probabilities respectively as *P*_*B*_, ${P}_{\varnothing }$, *P*_*C*_, *P*_*D*_, and *P*_0_. These probabilities depend on the mind-reading strategy adopted by an individual, as well as other continuous traits, determining features such as the inhibition of *C* responses, whose evolutionary dynamics we also explored. The fitness of a focal individual is determined by the sum of the average payoffs obtained as a solitary actor, *π*_+_, as an observer *π*_−_, or as an observed actor, ${\pi }_{\mp }$:

1 $$\pi =\mathrm{(1}-2p){\pi }_{+}+p\,{\pi }_{-}+p\,{\pi }_{\mp }.$$

We denote with the superscript • the elements related to a focal individual or strategy, while we use the superscript ° to indicate non focal, interacting individuals *y*°. For large populations, the behavior of *y*° approaches that of the average for all the strategies in the population. In the solitary actor case, the average payoff is simply ${\pi }_{+}={P}_{B}^{\bullet }\,b$. In the absence of any form of assortment, the average payoff as an observer is ${\pi }_{-}={P}_{D}^{\bullet }\,{d}^{-}-{P}_{C}^{\bullet }\,{c}^{-}.$ Similarly, the average payoff of an observed actor is ${\pi }_{\mp }={P}_{B}^{\bullet }\,b-{P}_{D}^{\circ }\,{d}^{+}+{P}_{C}^{\circ }\,{c}^{+}$. These probabilities depend on the cognitive strategies as described next.

### Mind-reading Strategies

We explore three main mind-reading strategies (Fig. 1), defined as the general organization of the cognitive strategy used by an observer to infer the best response *a*_−*i*_ in response to a social cue *s*_−*i*_. First, we implement an “innate” strategy, in which an observer responds to social cues with a fixed *s*_−*i*_ → *a*_−*i*_ mapping that adapts only through evolution, but not learning (Fixed Strategy - F). Second, a strategy in which social responses are only learned through social interactions (Associative strategy - P). In the third type, (Simulative strategy - S) an observer takes advantage of its own as-actor set of responses to infer actors’ behavior. These different cognitive strategies are built with two basic building blocks: representations and cognitive functions.

*Representations* correspond to the neural configurations and physiological responses associated with a stimulus or action, represented within clouds in Fig. 1. A stimulus representation ${\hat{s}}_{-i}\in \hat{S}$ is triggered by the perception of a stimulus, while an action representation ${\hat{a}}_{\mp i}$ describes the neural configuration and physiological state priming a certain action, and thus may trigger ${a}_{\pm }\in \hat{S}$. For simplicity, we summarize the complexities of a stimulus, its salience and the accuracy of perception and of the corresponding representations with a single variable *x*, defined as intensity. This can be seen as the extent of activation of the underlying neural configuration. Thus formally a representation is a pair, comprising the action or stimulus represented and an intensity, e.g. $\hat{s}=({s}_{\pm i},x)$.

*Cognitive functions*, represented in Fig. 1 as arrows, correspond instead to cognitive processes, determining the relationships between stimuli, actions and their representations, mapping from one space to another. For example, an actor perceives a stimulus *s* via a *perception function* (formally a stochastic map), that maps it to a stimulus representation $\hat{s}$. The actor learns to map $\hat{s}$ to an action $\hat{a}$ via a *learning function l*, and in turn performs an action via an *activation function α*:

2 $$s\in S\mathop{\to }\limits^{\gamma }\hat{s}\in \hat{S}\mathop{\to }\limits^{l}\hat{a}\in \hat{A}\mathop{\to }\limits^{\alpha }a\in A.$$

We assume that an individual’s behavior is only influenced by two variables in addition to its own strategy: the intensity of a stimulus and its own previous learning experience. Regarding the former we assume that the higher *x*, the higher the probability is that *l* would associate a correct action representation, and that an action would be performed. Learning, defined as the the reduction of the classification mistakes as experience is acquired, occurs in time *t* at a constant rate *λ*_*l*_. The specific mathematical expressions used to describe cognitive functions are reported in Table 2.

**Table 2:** Cognitive functions.

Function	Cognitive process	Probability
$\gamma :S\to \hat{S}$	*s*_±*i*_ → (*s*_±*i*_, *x*)	$P({\hat{s}}_{\pm i}|{s}_{\pm i})=\gamma$
$l(x,t):\hat{S}\to \hat{A}$	(*s*_±*i*_, *x*) → (*a*_±*i*_, *x*)	$P({\hat{a}}_{\pm i}|{\hat{s}}_{\pm i})=(1-{e}^{-{\rho }_{l}\cdot x}\mathrm{)(1}-{e}^{-{\lambda }_{l}\cdot {t}_{e}})$
$\alpha (x):\hat{A}\to A$	(*a*_±*i*_, *x*) → *a*_±*i*_	$P({a}_{\pm i}|{\hat{a}}_{\pm i})=(1+{e}^{-{\rho }_{a}(x-{u}_{B})/{u}_{B}}{)}^{-1}\mathrm{.}$
${\gamma }_{s}:{\hat{S}}^{-}\to {\hat{S}}^{+}$	(*s*_−*i*_, *x*) → (*s*_+*i*_, *uDx*)	$P({\hat{s}}_{+i}|{\hat{s}}_{-i})={\gamma }_{s}$
${\gamma }_{a}:{\hat{A}}^{+}\to {\hat{A}}^{-}$	(*a*_+*i*_, *uDx*) → (*a*_−*i*_, *uDx*)	$P({\hat{a}}_{+i}|{\hat{a}}_{-i})={\gamma }_{a}$

Each cognitive function can be mapped to specific probabilistic functions, that determines the probability of *D*, 0 and *C* responses for the different strategies. Hence the fitness of a strategy is simply given by the expectations *P*_*D*_, *P*_*C*_ and *P*_0_ of the cognitive functions organized as in Fig. 1. Cognitive functions depends on the following parameters: the probability that a stimulus is perceived correctly *γ*; a constant learning rate *λ*_*l*_; *ρ*_*l*_ describes how the intensity of a stimulus representation affects the accuracy of the responses. The activation function *α* inhibits irrelevant stimuli with intensity lower than a threshold *u*_*B*_, and produces an action when the intensity is higher. The higher *ρ*_*a*_, the higher is its discrimination power. We assume a sigmoid activation function^28,60–62^ so that *α*(*x* = 0) = 10^−6^ (hence *ρ*_*a*_ = 13.8155), so that no actions are performed in the absence of stimuli. *u*_*D*_ determines the intensity of simulated representations. Later in addition, we use a model with learning rate 1 and step linear activation. Note that while here specific analytical functions are assumed for the figures, we perform a general evolutionary invasion analysis of the model in section 2 of the Appendix.

Notice that actors encounter stimuli with probability 1 − *p*, while observers at a rate *p*. Hence, the scheme in Eq. 2 describes an actor, who perceives a stimulus *s*_+ *i*_ and responds with the best action *a*_+*i*_ with higher probability as its experience increases as (1 − *p*)*t*. The same also describes an observer, who responds to social cues *s*_−*i*_, choosing the best response *a*_−*i*_ with higher probability as its experience of social interactions increases as *pt*. We define this first type of observers/mind-reading strategy as P.

### Detailed description of simulative strategies

Besides social interaction, an observer has also its own experience as an actor to infer of what an individual would respond to a stimulus *s*_+ *i*_. Thus we contrast strategy P with a simulative strategy S, in which an observer takes advantage only of the information acquired as an actor, i.e. its own ${\hat{s}}_{+i}\to {\hat{a}}_{+i}$ mapping. Since this circuit leads, when the individual is an actor, to action *a*_+*i*_, a potential drawback of this strategy is that this action might be actually performed by the observer, resulting in a coordination event. This could be avoided if the underlying representation ${\hat{a}}_{+i}$ leading to *a*_+*i*_, is inhibited. In our model this inhibition is allowed to evolve (see Results and Appendix). To describe the recruitment of the as-actor network when having perceived a social cue ${\hat{s}}_{-i}$ we introduce two further cognitive functions, *γ*_*s*_ and *γ*_*a*_ (Fig. 1d): *γ*_*s*_ represents the probability of a correct association between an observed social cue and an as-actor stimulus $({\hat{s}}_{-i}\to {\hat{s}}_{+i})$; *γ*_*a*_ represents the probability of performing an appropriate social response, given a correct inference about the actor’s action $({\hat{a}}_{+i}\to {\hat{a}}_{-i})$. For simplicity the efficiency of these processes is described by fixed parameters named after the functions, *γ*_*s*_ and *γ*_*a*_. However, it is possible to devise different variations of the model. For example, *γ*_*a*_ could improve through learning (Table 2, Appendix 5). Finally, we contrast strategies P and S with a Fixed strategy (F), representing observers who employ a fixed mapping $\hat{s}\to \hat{a}$ optimized by evolution, rather than learning. Hence, their fitness depends entirely on the variability of the *Environment* (see below).

### Environment and learning

In the deterministic model we use a continuous-time model, where both learning and payoffs depend on the amount of time spent in a given interaction type. This corresponds to individuals experiencing a large number of interactions. Stochasticity in the amount of interaction, stimuli and in the learning process is addressed later in our evolutionary simulations, implementing computational agents with simple learning rules. Learning, and thus the efficiency of individuals’ responses, depends on the time *t* spent experiencing a given set of stimulus-action responses. The *environmental state* specifies the best response for each stimulus, and is thus represented by a mapping *S* → *A*. In a constantly changing environment with rate *λ*_*e*_ and individuals dying at a rate *λ*_*d*_ = 1, the time experienced by an individual in a given environmental state is distributed exponentially with rate *λ* = *λ*_*d*_ + *λ*_*e*_. For simplicity, we assume that environmental states are non correlated, so that individuals have to learn the appropriate reactions independently for each. Since strategies F rely on a fixed mapping, their fitness is proportional to the frequency of the most frequently visited environmental state. To be conservative in favor of F-strategies we assume that this is always visited once, so that it is the only experienced environment when ${\lambda }_{e}\ll {\lambda }_{d}$.

## Results

### Simulative strategies dominate in more complex environments

In our model a mind-reading strategy is determined by the mind-reading strategy-type and by a number of continuous traits, which affect the level of inhibition of coordination. We focus on two main questions: *(1) Under which circumstances are as-actor networks recruited for mind-reading? (2) Can emotional contagion evolve as a consequence of mind-reading?* The first question is addressed here by tracking the frequency of the different strategy-types, $F$, $P$ and $S$, in an infinite population. We address the second question in the next section, performing an evolutionary invasion analysis on the continuous traits influencing simulative strategies, ${u}_{D}$ and ${u}_{B}$.

**Figure 2:**
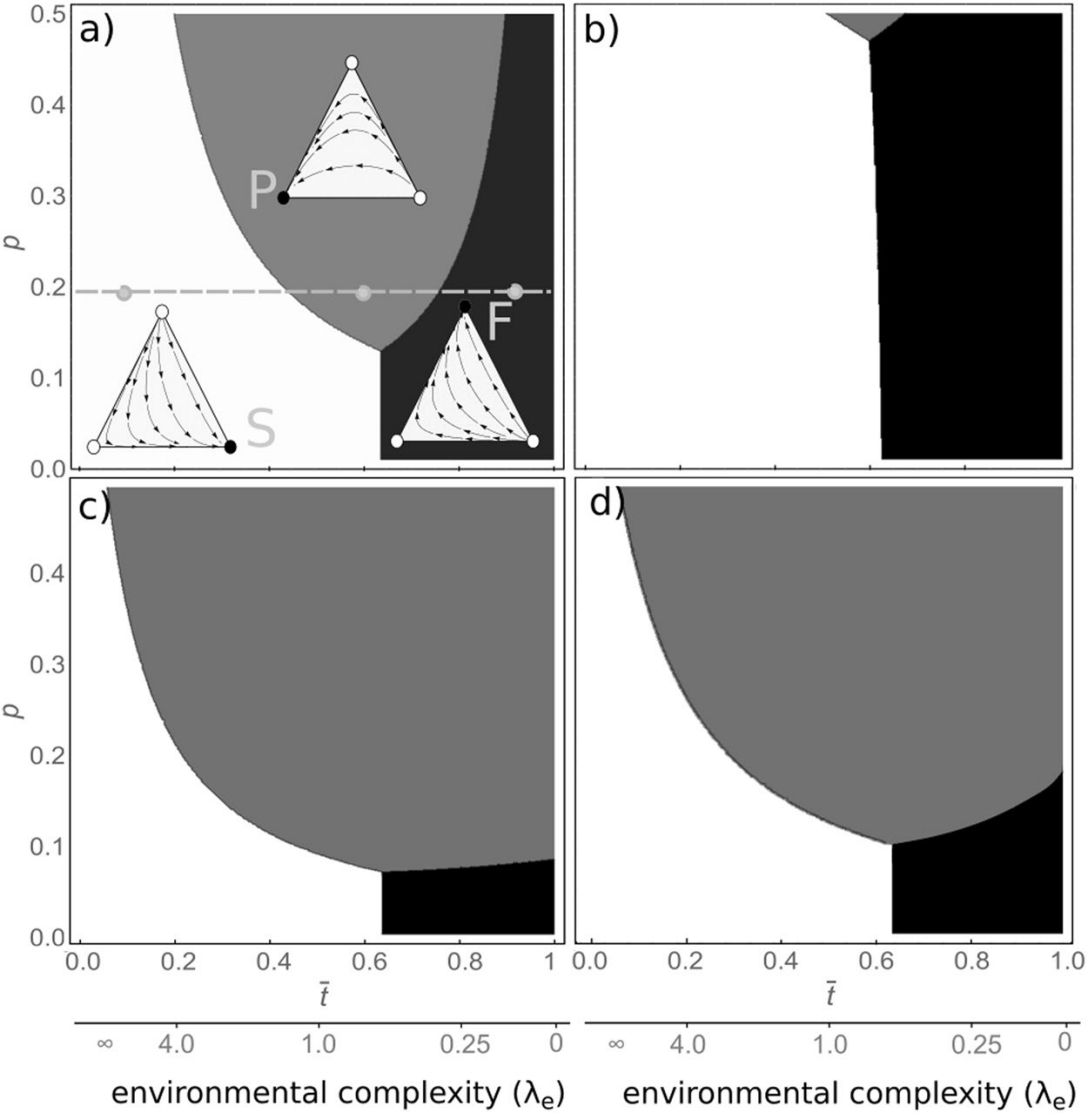
Evolutionary dynamics of the three mind-reading strategies, *F* (black), *P* (gray) and *S* (white), as a function of average time spent in a single environmental state ($\bar{t}\,=\,\mathrm{1/(}{\lambda }_{e}+{\lambda }_{d})$, x-axis) and the fraction of effective as-observer vs as-actor stimuli (*p*_−_, y-axis). Gray dots indicate the combinations of parameters for which the evolutionary dynamics are shown in the ternary plots, when $S$ (right vertex), *P* (top) or *F* (left) dominate. Here we consider a fixed *u*_*B*_, hence a single optimal *S*-strategy S^*^ exists, independently of the frequencies of other strategies. In (**a**) the parameters are *u*_*B*_ = 0.3, ${\rho }_{a}=13.8155/{u}_{B}$, normally distributed *x* with mean 0.5 and deviation 0.1, *ρ*_*l*_ = 5, *λ*_*d*_ = 1, ${d}^{-}={d}^{+}\mathrm{=5}$, ${c}^{-}={c}^{+}\mathrm{=3}$, ${\lambda }_{l}=50$. In (**b**) the learning rate is *λ*_1_ = 10, corresponding to the presence of 5 times more stimuli. In (**c**,**d**) individuals differ from each other in their responses with average probability *ψ* = 0.5. In addition, in (**d**) individuals interact twice as often with individuals with the same behavior.

First, we compute the expected probabilities of a B response as actor, and D and C as an observer using one of the three different strategies, indicated by the superscripts P, F and S. These determine the fitness of the mind-reading strategies, following Eq. 1. We report here expressions for the simplified case where every stimulus has intensity *x* = 1 and is sufficient for correct response given that an individual has enough experience, i.e. *α*(1) = 1 and $li{m}_{t\to \infty }\,l\mathrm{(1},t)={l}_{t}(t)=1-{e}^{-{\lambda }_{t}t}$. In this case the payoff of an actor is

3 $${\pi }_{+}={P}_{B}\,b=\frac{\lambda (1-p)}{{\lambda }_{d}+{\lambda }_{e}+\lambda (1-p)}b,$$

while the expression for the fitness of an observer depends on its strategy, indicated as superscript:

4 $${\pi }_{-}^{S}={l}_{x}({u}_{D}\mathrm{)((1}-\alpha ({u}_{D}))\,{\gamma }_{a}\,{d}^{-}-\alpha ({u}_{D})\,{c}^{-})\,{P}_{B}^{2}$$

5 $${\pi }_{-}^{F}=\frac{{\lambda }_{d}}{{\lambda }_{e}+{\lambda }_{d}}{P}_{B}\,{d}^{-},$$

6 $${\pi }_{-}^{P}=\frac{p{\lambda }_{l}}{{\lambda }_{d}+{\lambda }_{e}+p{\lambda }_{l}}{P}_{B}\,{d}^{-},$$

with ${l}_{x}(x)=1-{e}^{-{\rho }_{l}}$. Notice that the payoffs of all observers depend on the behavior of the observed actors ${P}_{B}^{\circ }$, but not on other observers. Thus, when *P*_*B*_ differs between individuals with different mind-reading strategies the evolutionary dynamics of this system are frequency-dependent. We explore this case in the appendix, where we show that different mind-reading strategies can be bistable. Here, we restrict to the simpler frequency-independent case, for which all main results hold. In this case, we can write the replicator dynamics of the system as:

7 $$\frac{d{y}^{j}}{dt}={y}^{j}({\pi }^{j}-\varphi )={y}^{j}({\pi }_{-}^{j}-{\varphi }_{-})$$

where *j* indicates one of the strategies *P*, *F* or *S*, and where $\varphi ={y}^{F}\,{\pi }^{F}+{y}^{P}\,{\pi }^{P}+{y}^{S}\,{\pi }^{S}$ is the average fitness, and ${\varphi }_{-}={y}^{F}\,{\pi }_{-}^{F}+{y}^{P}\,{\pi }_{-}^{P}+{y}^{S}\,{\pi }_{-}^{S}$ is the average of the as-observer components of the payoff. This system is characterized by transcritical bifurcations: each strategy dominates when its social fitness *π*_−_ is higher than those of other strategies. Thus, we can explore under which conditions the different strategies would evolve by simply comparing the expression for the social fitness in Eqs 4–6 (Fig. 2a). These expressions reflect the different sources of information that the three strategies use to cope with uncertainty, which we explore here in the form of environmental variability, behavioral complexity, and inter-individual differences.

In Fig. 2a we modify environmental variability through the parameter *λ*_*e*_, the rate of environmental change. Strategy F does not rely on learning, but on a fixed mapping optimized by evolution. Hence while this strategy is very efficient for predictable environments (low *λ*_*e*_), it cannot adapt to new environmental states. This is apparent in the term ${\lambda }_{d}/({\lambda }_{d}+{\lambda }_{e})$ in Eq. 5, that coincides with the fraction of time spent in a single predictable environmental state. Conversely, both strategy *P* and *S* use learning to adapt to new environments, and dominate when environmental variation increases. However, *P* learns only during social interactions, and the probability of successful *D* responses decreases linearly with *p*. For this reason, S evolves at lower values of *p*. Remarkably, *S* outperforms all other strategies as complexity increases. As information about the social context decreases, the experience gained as an actor becomes more valuable. This result is exemplified by simple analytical expressions obtainable in the simple case of an innate as-actor behavior. In this case the fitness of strategy *S* is independent of complexity, whereas the fitness of an observer using F or P strategies is ${d}_{-}\,{\lambda }_{d}/({\lambda }_{e}+\mathrm{1)}$ versus ${d}_{-}\,p\,{\lambda }_{l}/({\lambda }_{d}+{\lambda }_{e}+p{\lambda }_{l})$, respectively. Both of these decrease with environmental variability *λ*_*e*_, whilst that of strategy P also decreases with reduced opportunity of direct as-observer learning *p*. Therefore *S* -strategies dominate when environmental complexity increases. This pattern is consistent across the assumptions and models tested. In general, learning as an actor is faster than as an observer (supplementary material section 1.5.4), due to the uncertainty involved in social cues. Information about other individuals is inherently noisy or inaccurate, since an observer may not know what stimulus or internal state precisely characterize an actor, or simply might misperceive a social cue. Hence, in complex environments (high *λ*_*e*_) or when social stimuli are noisy and inaccurate (low *p*), using information acquired solely through social interactions may not suffice, requiring a large number of learning instances. Similar results are obtained when individuals can switch from *S* to *p* over their lifespan depending on the available information (Appendix).

The effect of behavioral complexity is manipulated by varying the learning rate, that we assume to be inversely proportional to the number of stimuli to be learned. As behavioral complexity increases, *S* strategies are favored (Fig. 2b).

We then explored the effect of inter-individual differences between individuals, that could arise because of differences in genetics, social status, or simply experience between the observer and the actor. These differences are detrimental to *S* strategies, since an *S* -observer infers the behavior of other individuals from its own, possibly extrapolating incorrect inferences if the two individuals differ in their responses to a given environmental stimulus, i.e. their own environmental state. We implemented these differences by using a probability *ψ* that individuals differ in the response to a particular as-actor stimulus (i.e. two different individuals would respond to a stimulus with two different optimal actions). *S*-observers have the possibility to make correct inferences only when the focal observer and the observed actor share the same behavioral response, and fail in a fraction *ψ* of the interactions. Thus, inter-individual differences impact negatively the fitness of *S*-strategies, reducing the range of parameters for which they would evolve (Fig. 2c). However, *S* -strategies still dominate in variable environments, and when *p* is low (Fig. 2c). This occurs because inter-individual differences do not only affect the fitness of *S* strategies, but also that of *P* strategies. In fact, only a fraction 1 − *ψ* of the individuals share the same response, and therefore the probability of encountering a specific stimulus-action is reduced by a factor 1 − *ψ*, requiring longer times to learn the behavior of the different individuals and mimicking the effects of environmental or behavioral complexity.

Note that all these results can be easily extended to cases in which individuals interact preferentially with some other individuals in a population, because of group structure, relatedness, or social status and dominance relationships. For example, individuals might interact more frequently with individuals with similar behavior. In this case, inter-individual differences will be partially masked, and exert a reduced effect on *S* -strategies, that in turn would be favored (Fig. 2d).

Concluding, as environmental complexity increases and self information becomes valuable, *S* strategies are able to invade even if this causes more coordination. In our simplified model, an upper bound for the probability of coordination is:

8 $$\frac{{d}^{-}}{{d}^{-}-{c}^{-}}\frac{{\lambda }_{d}+{\lambda }_{e}}{{\lambda }_{d}+{\lambda }_{e}+{p}^{-}{\lambda }_{l}} > {P}_{C}\,\mathrm{.}$$

### Accidental coordination is sustained at the evolutionary equilibrium

In our model coordination is costly for the observer. Eq. 8 establishes an upper bound to the amount of coordination that can be tolerated by a simulative strategy. We can now ask under what conditions will accidental coordination be present (*P*_*C*_ > 0) by looking at how inhibition evolves.

**Figure 3:**
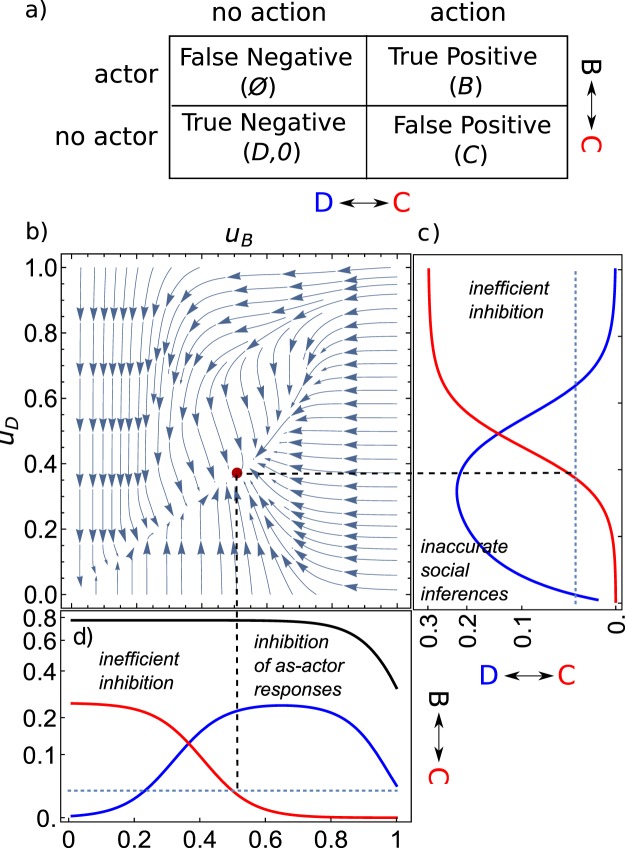
(**a**) Trade-offs constraining the recruitment of the as-actor network by simulative strategies. The as-actor network can be activated (left) or inhibited (right). For an actor (top), the inhibition of the as-actor network prevents an appropriate response B (False Negative), while if activated B can be performed (True Positive). When the individual is no actor but an observer (bottom), the activation of the as-actor network leads the observer to respond as an actor (C, False Positive). (**b**) Direction of the selection gradient for a resident population with traits *u*_*D*_, the intensity of simulation (x-axis), and *u*_*B*_, the inhibition threshold (y-axis). (**c**) Frequencies of *D* (*P*_*D*_, blue) and *C* (*P*_*C*_, red) responses, for a fixed value of ${u}_{B}={u}_{B}^{\ast }$. In this case *u*_*D*_ evolves maximizing the as-observer fitness. (**d**) *P*_*D*_, (blue) and *P*_*C*_ (red) and *P*_*B*_ (black) for a fixed value ${u}_{D}={u}_{D}^{\ast }$. A singular strategy occur when the sum of the as-actor component (proportional to *P*_*B*_) and as-observer component (a payoff weighted sum of *P*_*D*_ and *P*_*C*_) of the fitness is highest.

With efficient inhibition, an *S* -observer will be able to extract information from its as-actor network, without generating the response usually exhibited as an actor, here *C*. These cases can be seen as True-Negatives, as inhibition is applied correctly to avoid a costly and inappropriate as-actor response (here *C*, see Fig. 3a). This inhibition of the as-actor network can be achieved in different ways, that here we can categorize on the basis the types of errors that they might incur on, i.e. false positives and false negatives, and thus facing different evolutionary trade-offs. A first mechanism is to modulate how the as-actor network is recruited as an observer, potentially affecting the accuracy of social inferences: the more the as-actor network is allowed to take over, the more accurate the simulation; however, the chance that an as-actor action is triggered increases, potentially leading also to an increased risk of coordination (*P*_*C*_, false positives, see Fig. 3a,c) together with accurate inferences (*P*_*D*_, true negatives). A second mechanism is to modify the structure of the as-actor network itself to make it easier to inhibit; however this would potentially lead to accidental inhibition of actions when the individual is an actor (false negatives, see Fig. 3a,b), decreasing *P*_*B*_ as well as *P*_*C*_.

In the following, we model these two general inhibition mechanisms as two one-dimensional continuous traits, *u*_*D*_ and *u*_*B*_ respectively. Of these, *u*_*D*_ can be seen as the intensity of the recruitment of the as-actor network in the as-observer context, or the extent of shared representation. The second, *u*_*B*_, can be seen as an activation threshold, inhibiting as-actor responses when the activation of the as-actor network is below the threshold. In the discussion we address the generality of results from this simple model.

When *u*_*B*_ is fixed, *P*_*B*_ is identical for all individuals, thus the system is frequency independent. In this case, the fitness of a mutant strategy *S* with *u*_*D*_ value ${u}_{D}^{m}$ is independent of the fitness of the other *S* strategies present in the population. Therefore, a single *S* strategy dominates and reaches fixation, leading to an evolutionary equilibrium value ${u}_{D}^{\ast }$, for which the invasion fitness $\frac{d{\pi }_{-}^{S}({u}_{D}^{\ast })}{d{u}_{D}}$ equals 0. This is the global maximum of the function ${\pi }_{-}^{S}({u}_{D})$, and is both an evolutionarily stable strategy (ESS) and convergence stable (CS).

We see that in most cases this equilibrium is internal, i.e. *u*_*D*_ > 0, leading to a small level of accidental coordination (0.5 − 2% of the interactions in the examples tested), even though inhibition is allowed to evolve and other strategies to be used (Fig. 3b–d). We perform a general evolutionary invasion analysis in section 2 of the Appendix, where we identify the conditions required for the evolution of an internal equilibrium with accidental coordination. However, to explain intuitively why this occurs, we obtain here analytical expressions for ${u}_{D}^{\ast }$ by assuming specific expressions for the cognitive functions $\alpha ({u}_{D},\,x)$ and $l({u}_{D},\,x,\,t)$ in Eq. 4. In particular, we explore the simplified case of stimuli with identical intensities equal to 1, with step-linear dependencies on the representation intensities for the activation/inhibition and the learning function:

9 $$l=\{\begin{array}{ll}x & 0\le x\le 1\\ 1 & x > 1\end{array},\,\alpha (x,{u}_{B})=\{\begin{array}{ll}0 & 0\le x\le {u}_{B}\\ {\rho }_{a}(x-{u}_{B}) & {u}_{B}\le x\le {u}_{B}+\mathrm{1/}{\rho }_{a}\\ 1 & x > {u}_{B}+\mathrm{1/}{\rho }_{a}\end{array}$$

In this simplified case, a full inhibition of accidental coordination would occur if ${u}_{D}^{\ast }\le {u}_{B}$, whereas accidental coordination occurs if ${u}_{D}^{\ast } > {u}_{B}$, the higher *u*_*D*_ the more coordination. When *u*_*D*_ <  *u*_*B*_ evolution always leads to higher values of *u*_*D*_, whereas when ${u}_{D}\le \mathrm{(1}+{u}_{B}{\rho }_{a})/{\rho }_{a}$ it leads to lower values, i.e. the invasion fitness is respectively always positive $(\frac{d{\pi }_{-}^{S}({u}_{D})}{d{u}_{D}}={\gamma }_{a}\,{d}^{-})$ or always negative $(\frac{d{\pi }_{-}^{S}({u}_{D})}{d{u}_{D}}=-\,{c}^{-})$. For intermediate values $({u}_{B}\le {u}_{D}\le (1+{u}_{B}{\rho }_{a})/{\rho }_{a})$ instead, a singular point satisfies:

10 $$0=-\,2{\rho }_{a}({\gamma }_{a}\,{d}^{-}+{c}^{-}){u}_{D}^{\ast }+{u}_{B}\,{\rho }_{a}({\gamma }_{a}\,{d}^{-}+{c}^{-})+{\gamma }_{a}\,{d}^{-}.$$

Thus, ${u}_{D}\ast$ the evolves towards the maximum of the as-observer fitness:

11 $${u}_{D}^{\ast }=\,{\rm{\max }}({u}_{B},\frac{1}{2}({u}_{B}+\frac{{\gamma }_{a}}{{\rho }_{a}}\frac{b}{b+c})).$$

Since accidental coordination evolves when ${u}_{D}\ast  > {u}_{B}$, this indicates that accidental coordination can evolve even if costly for the observer, provided that ${\rho }_{a} < \frac{{\gamma }_{a}}{{u}_{B}}\frac{b}{b+c}$. This condition indicates that coordination can be fully inhibited only if 1/*ρ*_*a*_ is high enough, i.e. the slope of the inhibition function is very steep and discriminative (Appendix, Fig. S1). Thus, this simplified case shows that evolution will lead to higher values of *u*_*D*_ - and in turn accidental coordination to increase - as long as the benefit of more accurate inferences outweighs the cost of a higher risk of accidental coordination events, that here increases proportionally to 1/*ρ*_*a*_ at increasing values *u*_*D*_ (Fig. 3c).

Remarkably, accidental coordination can evolve even when also the activation threshold can evolve, alone (Appendix, section 2.2) or simultaneously with *u*_*D*_ (Appendix, section 2.3). To see why, note that whenever *u*_*B*_ evolves to values higher than $1-{\rho }_{a}{u}_{D}$, also actions performed as an actor can be inhibited (Fig. 3d). This behavior is a specific example of a more general trade-off between true negatives and false positives: increasing the strength of inhibitory mechanisms can result in the unspecific inhibition of actions that could be advantageous for the observer. In particular, in the simplified case, the equilibrium value of *u*_*B*_, i.e. how selective the activation threshold is, decreases proportionally to (1 − *p*)/*p*, the odds of a potential false negative (Appendix section 2.2).

An important biological note is that this simplified model is likely conservative. First, also other as-observer actions could risk of being inhibited. Second, in real life scenarios, stimuli and the activation of neurophysiological pathways are also subjected to noise and variability. This uncertainty increases the chance of false negatives and false positives, and thus likely lead to scenarios that could be represented with smoother, more continuous activation functions, compared to the simplified model presented above. In these cases - that can be represented mathematically with a continuous activation function (e.g. a sigmoid) or normally distributed stimuli intensities - a small probability of accidental coordination always evolve (Appendix, section 2).

Finally, note that these results are not dependent on the specific functions used for the simplified model, and they reflect general payoffs underlying the trade-off between increasing the amount of true positives and negatives (appropriate responses B and D, respectively), and the risk of false poositives (C) and false negatives (unspecific inhibition of other actions, $\varnothing$). Thus, we show a generalization of these results in section 2.1–2.3 of the Appendix. Furthermore, we show that these results hold even for more complex models of inhibition and social responses (section 2.4 and 4 of the Appendix).

### Kin selection and indirect benefits of coordination

We also investigated the interaction between simulative strategies and kin selection. To this aim, we modeled the indirect fitness benefits of kin selection by adopting a payoff structure similar as in Taylor and Nowak^22^, using *r*, an abstract assortment coefficient that can represent either relatedness or group structure^22^. We focus on the evolution of *u*_*D*_, the intensity of the recruitment of the as-actor network. The transformed payoff structure is shown in Table 3.

**Table 3:** Payoff matrix in presence of relatedness.

actor	*B*(*a*_+*i*_)	${\boldsymbol{\varnothing }}$(*a*_*j*≠+*i*_)
observer		
$D\,({a}_{-i})$	(*b* − *d*^+^) + *rd*^*−*^	0
	*d*^*−*^ + *r*(*b* − *d*^+^)	0
$0\,({a}_{j\ne \pm i})$	*b*	0
	*rb*	0
$C\,({a}_{+i})$	(*b* + *c*^+^) − *rc*^*−*^	0
	−*c*^*−*^ + *r*(*b* + *c*)	0

All payoffs are normalized by a factor 1 + *r*.

Notice that as *r* changes, the optimal strategy for the observer, which we called *D*, could change. For simplicity we first ignore this effect, assuming that *D* does not change with *r*.

Empirical evidence suggests that empathy, contagious yawning and emotional contagion are stronger with kin or in-group members, a phenomenon defined as *empathic gradient*. In our model, this would correspond to higher levels of recruitment of the as-actor network (*u*_*D*_) and coordination (*P*_*C*_) at increasing *r*. Therefore we investigate the effects of *r* on the recruitment of the as-actor network, *u*_*D*_. Our purpose is to estimate the effect of *r* on the evolutionary equilibrium for ${u}_{D}^{\ast }$, hence $\frac{d{u}_{D}^{\ast }}{dr}$.

**Figure 4:**
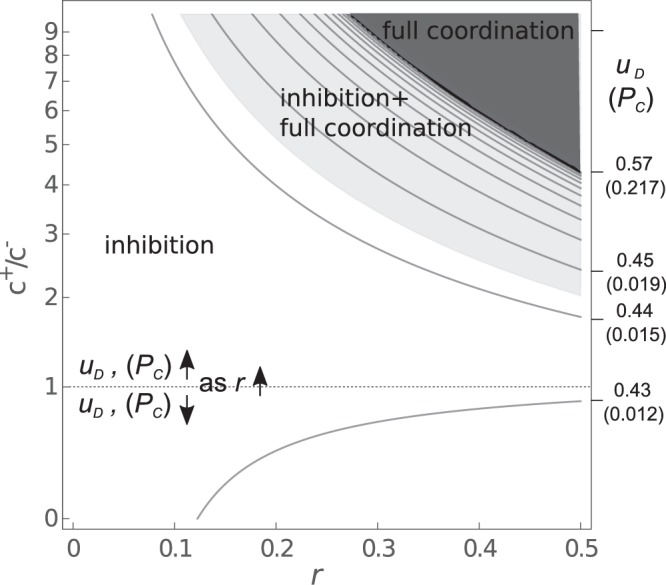
Effects of relatedness (x-axis) on the intensity of simulation *u*_*D*_ (level curves, top values) and on the probability of coordination (bottom values, in brackets), at varying payoffs. Payoffs are changed by varying the ratio of coordination payoff for actor and observer ${c}^{+}/{c}^{-}$, when ${d}^{+}/{d}^{-}$ is constant. Level curves indicate regions with different values of *u*_*D*_ for the internal equilibrium (inhibition), when this exists. The shaded area indicates regions of the parameter space where full coordination (*P*_*C*_ = 1) is evolutionarily stable. This can overlap regions of the parameter space where an internal equilibrium is also stable (inhibition + full inhibition), or not (full coordination, top right corner, in dark gray). In this plot we considered a sigmoid activation function and ${d}^{-}={d}^{+}\mathrm{=5}$ and ${\gamma }_{a}\mathrm{=1}$.

This can be obtained by implicit differentiation of the invasion fitness^23^ (see Appendix). We show that the conditions necessary to an *empathic gradient* analogous to what observed empirically are that *c*^+^/*c*^−^ > *d*^+^/*d*^−^. Note that the effect of relatedness on coordination is non-trivial: *r* can either increase (Fig. 4, high values of *c*^+^/*c*^−^) or even decrease *u*_*D*_ and *P*_*C*_ (Fig. 4, low values of *c*^+^/*c*^−^). An increase is observed provided that a more efficient mind-reading, i.e. an higher proportion of *D* responses, provides a substantial benefit, either direct and aimed at defection (*d*^*−*^) or cooperation (*rd*^+^). Hence a benefit of mind-reading might explain observed behavioral patterns of emotional contagion and empathy. The patterns described above are even stronger when *γ*_*a*_ is small (Appendix).

We see that for high relatedness, when cooperation is evolutionary advantageous, inhibition can go to 0 and the observer fully coordinates (Fig. 4, dark gray area). This effect could be called empathy–the observer does not inhibit simulated responses towards kin. However, one should remember here that we assumed that *D* is the same for higher values of *r*, while in this context it is of course possible that another strategy *D*_*r*_ other than *C* or *D* would be optimal for cooperation. In this case our framework can be applied without changes: an observer would simply use simulative strategies to infer the actor’s response and the best action to cooperate, while inhibiting coordination. Notice that for some intermediate values of *r*, full coordination and an internal equilibrium where coordination is inhibited can can be bistable (Fig. 4, light gray).

## Discussion

### Simulation is evolutionarily advantageous in complex environments

We have shown that private as-actor information, recovered through a partial recruitment of as-actor neural circuits, confers an important selective advantage in complex social contexts, when information on other individuals’ behavior is limited. Under these circumstances, simulative strategies evolve. A side effect of simulative strategies is that an observer could accidentally perform the same as an actor, a response that we define here as accidental coordination. We showed that even when this accidental coordination occurs, the fitness benefit of making more accurate inferences about others can buffer its costs. It has been questioned whether mirroring processes have evolved in the context of mind-reading, or simply emerged as by-products of general domain associative learning^24^. Here we have shown that alternative strategies, which rely only on information acquired as observer, are often outperformed by strategies relying on as-actor experience. An efficient mind-reading has a significant impact on the fitness of an individual. Thus it is unlikely that such an important process has not been subjected to the forces of evolution^25^. We have also observed that selective constraints act on inhibitory mechanisms of the as-actor neural network, avoiding action interference and modulating accidental coordination. Therefore even if associative learning plays a key role in the development of mirroring, selection is expected to shape the modulation of “shared representations”. This is supported by the evidence that a dysfunctional inhibition is pathological.

Brain regions involved in mind-reading and action or emotion recognition are often grouped in mentalizing ones (e.g. TPJ, dmPFC) and mirroring or empathy related-regions, also recruited as actors (e.g. somatosensory cortex, premotor and inferior parietal cortex)^21,26^. Both these circuits likely operate together in most real life tasks. Our model shows in which circumstances the latter ones might be beneficial and favored for mind-reading, by complementing the lack of social information, Clearly, in many instances learned information is abundant and a recruitment of the as-actor network is not necessary. Our model shows that this happens if enough social information is available, when enough learning opportunities are available and in easily predictable contexts.

### Evolutionary trade-offs hinder a complete inhibition of coordination

We have shown that small probabilities of coordination can be maintained even when costly, and when inhibition is allowed to evolve (Fig. 2a). The model suggests that two evolutionary trade-offs might hinder the efficiency of inhibitory mechanisms of contagion: In the first trade-off (D-vs-C, captured by trait *u*_*D*_, Fig. 3a) important as-actor information might be retrieved only by faithfully recruiting the as-actor neural network. Partially renouncing the information stored in as-actor circuits by inhibiting them upstream might decrease the accuracy of the simulative process itself and in turn that of social inferences^27^ (Fig. 3c). Hence, excessively reducing the recruitment of the as-actor network in order to avoid accidental coordination is disadvantageous. Note that the only underlying requirement for the emergence of this trade-off is that more intense/accurate stimuli representations would lead to more intense/accurate response. This requirement is biologically plausible and a common feature of models of neurophysiological responses^28^, besides being supported by empirical evidence^29,30^. A challenge for future research is to assess the validity of this principle in the context of simulative strategies. Note however that different studies already provided evidence that a reduced or impaired recruitment of the as-actor network might result in reduced social inferences. For instance, a pathologically impaired activation of the amygdala results in an impaired ability to infer disgust of fear in other individuals^31^. In addition, this might be exemplified by phenomena like motor interference (in which the intensity/accuracy of the recruitment of the as-actor network is affected by another stimulus), and pathologies such as alexithymia and autism, which affect the access to self representations of internal states and in turn perspective taking^4,26,31–33^. More studies apt to investigate the role of mirror neurons in action recognition could provide further evidence in support of this assumption.

The second trade-off (B-vs-C, captured by trait *u*_*B*_) is that a downstream mechanism inhibiting coordination must be able to inhibit the actions driven by internal neural configurations (or motivational states) that are usually experienced as actors, when certain alternative responses are instead advantageous (Fig. 3b). Such an inhibitory mechanism has to be extremely specific for the as-observer context, any non-specificity hindering its evolutionary advantage. One can ask if this is realistic. Why would an organism not be able to conditionally inhibit a particular response in a social context? Here, it is important to remember that its own decision making machinery is being used as a “black box”, and that is where the gain in using the simulative strategy comes from: the inferred responses are not known prior to the simulation. This makes completely specific inhibition relatively hard to achieve, since the downstream responses are not known a priori, without sufficient information. In particular, the autonomous and more peripheral components of behaviors with a strong emotional^7^ and physiological^16^ valence might be particularly hard to inhibit efficiently, as in contagious distress. Vice versa, a complete inhibition might be achievable when sufficient social information is available, as we show with a temporal model of inhibition (Appendix, section 2.4) and it is documented empirically^34–36^.

Note that for didactic purposes we presented these trade-offs in terms of simple evolvable traits (*u*_*D*_ and *u*_*B*_), each corresponding to a specific characteristic of a cognitive process. However, the trade-offs that we identified are not determined by the specific implementation or the specific cognitive functions used, as shown by a general evolutionary invasion analysis of the model (Appendix section 2). For example, although we presented *u*_*B*_ as the threshold of an activation function, a trade-off between the inhibition of coordination (true negatives) and undesired unspecific inhibition (false negatives, B-vs-C trade-off) would emerge for any form of inhibition, with increasing importance the higher the chance of false negatives. This is particularly relevant for the simulative ‘black-box’: even though an observer would like to inhibit coordination, the specific action to inhibit is unclear before this is inferred via simulation.

Despite this trade-offs, we have shown that coordination is inhibited efficiently in most cases and occurs only occasionally: in 0.1–2% of the total interactions in our model. Hence simulation is relatively efficient, despite leaving room for contagion under conditions with intense above-threshold stimuli or cheap coordination events, where inhibition through learning might not be reinforced e.g. contagious yawning. Thus, although rare, we suggest that accidental coordination should be considered in null models of social interactions. If a simulative strategy is used for mind-reading, the null behavior of organisms should not necessarily be ‘no action’, but accidental coordination. This model supports a parsimonious bottom-up explanation for the emergence of contagion and rudimentary forms of empathy, without requiring ad-hoc explanations for each specific contexts, e.g. e.g. contagious yawning^37^. Such explanation is consistent with the widespread presence of mirroring processes and related forms of coordination, facilitation or contagion, in social cognition.

### Empathy: mind-reading in cooperative contexts

Therefore, our model provides two non-mutually exclusive explanations for the evolution of emotional contagion and empathy, i.e. cooperation mediated by the recruitment of as- actor circuits. To illustrate them, we take the example of empathy towards an actor subjected to a painful or stressful stimulus.

First, the as-actor network might be simply recruited by a simulative observer to infer the state of an actor, in a context in which cooperation is evolutionarily advantageous for the observer as well as the actor, e.g. a simulative observer infers the state of a related actor and cooperate.

Second, when coordination is advantageous for the actor but not evolutionarily advantageous for the observer, we have shown that cooperation might still occasionally occur because of a missed inhibition of coordination from a simulative observer. For example, contagious distress can inhibit a conditioned response if this causes pain in another individual^38,39^, and in primates, grooming relieves the distress of the groomer, as well as the groomee^40,41^. Hence contagious distress might trigger in the observer a typical helping behavior like grooming, or hinder an action that is painful for the actor. A similar proximate mechanism is aversive-arousal reduction^42,43^: cooperation occurs when similar emotional responses are induced in an observer, who in turn helps the actor in order to relieve its own perceived distress. This phenomenon likely explains only a small fraction of seemingly altruistic interactions^44^, consistent with our predictions of a low probability of accidental coordination. Nevertheless, it is of a major theoretical interest since no ultimate mechanism promoting cooperation such as synergistic payoffs^18^ or assortment mechanisms (e.g. kin or group selection) is required^45,46^.

We suggest that the two mechanisms could be phylogenetically related. Simulative strategies, by recruiting the as-actor network, provide a simple proximate mechanism to synchronize the actions of an observer to the internal and motivational states of a recipient individual. Our model shows that the recruitment of the as-actor network and coordination increase when cooperation is promoted by assortment. Thus simulative strategies, evolved initially for mind-reading, could be have been later coopted for empathy and cooperation. A potential criticism is that a coordination of internal states and emotional responses is not sufficient, and may even hinder, cooperation. For example, it has been observed that contagious distress might undermine helping behavior^44,47^. However, we showed that coordination can evolve even when it is not the best possible cooperative response: coordination offers an evolutionary compromise, by synchronizing the internal state of an observer with that of an actor, and reducing the degrees of freedom for possible responses. For example, the perception of distress in the offspring elicits alertness in parents. From this rudimentary form of state-matching empathy, the activation of shared representations of pain or distress could have evolved to elicit prosocial responses, concurrently with the role of oxytocin in reducing avoidance responses triggered by distress^47^. This is supported by the evidence that the activation of stress-related responses in the observer often accompanies empathy and cooperation (e.g.^48,49^).

It has been proposed that parental care played a key role for the evolution of empathy. Our model is consistent with this hypothesis, since parental care would provide both a selective pressure for cooperation and the understanding of the needs of the offspring^39,42,47^. However, mind-reading benefits are not restricted to parental care, and in the spectrum of social complexity, parent-offspring interactions are relatively simple: because of their limited interaction with the environment, the behavior of offspring is mostly evolved as opposed to learned. Accordingly, instances of parental care exist throughout the animal world even in the absence of apparent empathy (e.g. eggs brooding, ritualized parental cares^50^). Thus, even though we cannot rule out that in mammals parental care provided the sufficient complexity to render simulative strategies adaptive, we suggest that simulative strategies might have evolved for mind-reading in a more general social context. Only later, might kin selection have coopted them for cooperation^21,27,51^, or emotional contagion when coordination is advantageous. These conclusions are supported by neurophysiological evidence, showing that the recruitment of the as-actor network extends beyond the cooperative context^4,12^; and behavioral studies, showing that contagion in non-cooperative context (e.g. contagious yawning, facial mimicry) is stronger with kin and unrelated but socially close individuals, with whom empathy and helping behavior (e.g. consolation^21,52^) are stronger^53–57^.

### Future perspectives

Our model suggests potential directions for new empirical studies, that would allow to test some of its predictions. For example, we predict that simulative strategies are more advantageous in unpredictable environments. While empirical evidence seems to support the presence of empathy especially in primates and other mammals, a better characterization of the species in which empathy is present would improve this picture. In addition, more studies are needed both at the intra-specific and inter-specific level to assess whether perspective-taking skills correlate with emotional contagion and empathy driven cooperation. Do species or individuals who simulate more also show more empathy driven cooperation?

We also showed that specific features of cognitive processes involved in mind-reading would be necessary and favour the evolution of accidental coordination. Thus, more studies would help to clarify when these conditions apply. For instance, we showed that accidental coordination is favored when inhibitory mechanisms incur the risk of false positives - inhibiting off-target actions. Thus, studies investigating the specificity of inhibitory mechanisms would be precious to clarify the role of the B-vs-C trade-off. Furthermore, more studies would be beneficial in assessing the role and the extent to what as-actor network circuits are necessary for inferences, for example in the case of mirror neurons. Concluding, the characterization of inhibition mechanisms and their specificity at the neurophysiological level will certainly help to understand whether and to what extent accidental coordination contributes to prosocial behavior in different species.

## Conclusions

Our model explores a simple assumption, that experience as actor can be useful to predict and interpret others’ actions, and shows that this has relevant implications and side-effects. First, we show that strategies relying on this source information can evolve under many circumstances. Second, we show that a side effect of the evolution of such strategies is that an observers could fail to inhibit the observed behavior coordinating with the state and actions of the observed actors. These two phenomena provide us with a simple unified perspective on biological phenomena as different as mirror neurons, motor interference, contagious yawning, contagious distress and empathy, and their higher stronger effects with kin. Many other biological pathological and non pathological behaviors could possibly be connected. For example, the comorbidity of alexythymia and autism had already been interpreted as the inability to understand others as a consequence of the impaired awareness of one’s own feelings^4,26,31–33^. The fact that information and neural circuits acquired as an actor are used to interpret others’ actions is also apparent for sensory stimuli in the activation of the somatosensory cortex^26^; for stimuli of different nature in phenomena like mirror touch synesthesia; and possibly even for collective inferences and abstract concepts^20,58^ and for inanimate or inter-specific entities (e.g. anthropocentrism^59^). Furthermore, the role of inhibition of the activation of the as-actor network to interpret others’ actions is apparent in pathologies like echopraxia and echolalia^14^. As an actor’s cognition is embodied, even an observer’s cognition is required to be embodied, despite the risk of accidental coordination^10^. Concluding, the importance of information acquired as an actor, either learned or evolved, is pivotal to understand social cognition and its evolution.

## Supplementary information

Appendix
