# Supplementary material for: The complexity of understanding others as the evolutionary origin of empathy and emotional contagion
Source: Sci Rep. 2019 Apr 8;9:5794. doi: 10.1038/s41598-019-41835-5 (PMC6453980; doi:10.1038/s41598-019-41835-5)
Supplement: Supplementary file 1 — Appendix [file 41598_2019_41835_MOESM1_ESM.pdf]

The complexity of understanding others as the evolutionary origin of empathy and emotional contagion

Fabrizio Mafessoni[1]*, Michael Lachmann[2]

[1] Max Planck For Evolutionary Anthropology, Leipzig, 04103, Germany

[2] Santa Fe Institute, Santa Fe, New Mexico,87501, USA

*Corresponding author: fabrizio.mafessoni@gmail.com

Appendix

Below we provide additional information on the model following the same notation of the main text, summarized in Table S1. First we provide additional details on the cognitive strategies and how fitness expressions are obtained. Second, we show how the results presented in the text are obtained in detail. Last, we present agent based simulations testing the model in presence of simple learning rules. In particular:

- Section 1. Deterministic model, detailed description:
  - 1.1 General expressions for fitness
  - 1.2 Cognitive strategies
- Section 2. Evolutionary analyses:
  - 2.1 u_D evolvable.
  - 2.2 u_B evolvable.
  - 2.3 u_D and u_B evolvable.
  - 2.4 Temporal inhibition (u_S evolvable).
- Section 3. Kin selection
- Section 4. Simulation with as-observer learning
- Section 5. Agent Based Evolutionary Simulations.

Section 1

General expressions for fitness

We denote the strategy an individual with $\mathbf{u}$. The strategy that an individual adopts is defined by a strategic type (F, P or S), and one or more continuous traits, denoted with the letter u, i.e. u_D, u_B and u_S. The strategy of an individual determines the probability of a given response to a stimulus: B or $\emptyset$ if the individual is an actor, D,C and 0 if an observer. We denote the probabilities of a given response in the form $p(x, t, \mathbf{u})$, dropping the arguments for simplicity when not necessary. For instance for an actor, $p_B(x, t, \mathbf{u}) = \Pr(a_{+i} \,|\, s_{+i};\, x;\, t)$ defines the probability of an appropriate B response. A focal observer reacts appropriately to a social stimulus with probability $p_D(x, t, \mathbf{u}) = P(a_{-i}|s_{-i}; x; t; \mathbf{u})$.

Symbol	Definition
s	Stimulus. It can be perceived as an actor (e.g. s_{+i}) or as an observer (e.g. s_{-i})
a	Action. The best response for a stimulus s_j is a_j
x	Intensity of a neural configuration (e.g. the perception of a stimulus)
t, t_+, t_-	Absolute time, time that an individual interacts as an actor and as an observer.
B	Best response for an actor (e.g. a_{+i} in response to s_{+i}).
D	Best response for an observer (e.g. a_{-i} in response to s_{-i}).
C	Coordination. It occus if an observer performs a_{+i} in response to s_{-i}).
$0, \emptyset$	Failed action (e.g. a_{+j} in response to s_{+i}), for observers and actors, respectively.
$\mathbf{u}$	Strategy, defined by the strategic type (F,P and S) and traits like u_D and u_B.
u_D	Intensity of simulation. If $u_D = 0$ the as-actor network is fully inhibited.
u_B	Threshold of activation of an action. The higher, the easier the inhibition
Cognitive functions	Mappings from stimuli and actions to representations. Described in Table 2.
$\bullet$	Focal individual/strategy
$\circ$	Non-focal individuals/strategies
$\pi_+, \pi_-, \pi_\pm$	Payoff as actor, observer and observed actor
p_Y	Probability of a given response Y for a specific stimulus
P_Y	Average probability of a given response Y for a given strategy
$\lambda_l, \lambda_e, \lambda_d$	Learning, death and environmental change rate
$p_- = p, p_+ = (1-p)$	Probabilities that an individual is either an observer or an actor.
r	Assortment of relatedness coefficient
D_r	Cooperative response, providing a benefit b^+ to an actor at a cost $-b^-$ for the observer.

Similarly, we can define the probability that it reacts coordinatively with $p_C(x, t, \mathbf{u}) = \Pr(a_{+i} | s_{-i}; x; t; \mathbf{u})$, which leaves $p_0(x, t, \mathbf{u}) = \Pr(a_{j \neq \pm i} | s_{-i}; x; t; \mathbf{u}) = (1 - p_D(x, t, \mathbf{u}) - p_C(x, t, \mathbf{u}))$. We can now express the expectations of a given response in terms of p_B, p_D, p_0 and p_C. For a focal individual $\bullet$, the expected probability of B responses is simply

$$P_B^\bullet = E[p_B^\bullet] = E_{x^\bullet, t^\bullet}[p_B(x^\bullet, t^\bullet, \mathbf{u}^\bullet)] \,. \tag{1}$$

We use the notation E_y to indicate the expectation over the environmental variable y. For brevity the expectation over all the possible variables, for actors and observers, is indicated by E, without further subscripts. P_B° can be calculated by averaging also across all the population strategies (i.e.

$P_B^\circ = E_{x^\circ,t^\circ,\mathbf{u}^\circ}[p_B(x^\circ,t^\circ,\mathbf{u}^\circ)] = E[p_B^\circ]$). For social interactions we have in the most general form:

$$P_D^\bullet = \quad E[p_D^\bullet\, p_B^\circ] = E_{x^\bullet,t^\bullet,x^\circ,t^\circ,\mathbf{u}^\circ}[p_D(x^\bullet,t^\bullet,\mathbf{u}^\bullet)\, p_B(x^\circ,t^\circ,\mathbf{u}^\circ)]\,, \tag{2}$$

$$P_C^\bullet = \quad E[p_C^\bullet\, p_B^\circ] = E_{x^\bullet,t^\bullet,x^\circ,t^\circ,\mathbf{u}^\circ}[p_C(x^\bullet,t^\bullet,\mathbf{u}^\bullet)\, p_B(x^\circ,t^\circ,\mathbf{u}^\circ)]\,, \tag{3}$$

$$P_0^\bullet = \quad 1 - P_D^\bullet - P_C^\bullet\,, \tag{4}$$

where analogous expressions can be obtained for P_D°,P_C° and P_0°.

Note that $t^\bullet$ or t°, as well as $x^\bullet$ and x°, are not necessarily independent, and their relationships will depend on the specific structure of the environment. For example, the intensity of a stimulus perceived by an actor, x_+, and by an observer, x_-, might be correlated. Similarly, environmental states can vary independently for all individuals(asynchronous/spatial variation, $t^\bullet$ independent of t°), or at the other extreme, vary simultaneously (synchronous/temporal variation, $t^\circ = t^\bullet$).

We observed that differences in these correlations do not lead to any qualitative difference in the behavior of the model, even in the most extreme cases. Thus in agent-based simulations we adopt a synchronous variation and discrete generations setup for computational efficiency. In the deterministic description of the model we report general expression that can be applied to all cases, while for figures we assume asynchronous variation and uncorrelated intensities of actors' and observers' representations of a stimulus, i.e. independent $t^\bullet$ and t°, and independent $x^\bullet$ and x°. In this case eq.S2-3 become:

$$P_D^\bullet = \quad E[p_D^\bullet\, p_B^\circ] = E[p_D^\bullet]\, E[p_B^\circ], \tag{5}$$

$$P_C^\bullet = \quad E[p_C^\bullet\, p_B^\circ] = E[p_C^\bullet]\, E[p_B^\circ]. \tag{6}$$

For readability, later we also report simplified expressions obtained under the assumption that all stimuli intensities are identically distributed and equal to 1, i.e. $x_+ = x_- = 1$.

Cognitive strategies

Cognitive strategies can be represented using the functions defined in the main text and reported in Table 2. We also obtain simplified expressions by using the step linear functions:

$$l_x = \begin{cases} x & 0 \le x \le 1 \\ 1 & x > 1 \end{cases}\,, \qquad \alpha(x,u_B) = \begin{cases} 0 & 0 \le x \le u_B \\ \rho_a\,(x - u_B) & u_B \le x \le u_B + 1/\rho_a \\ 1 & x > u_B + 1/\rho_a \end{cases}\,. \tag{7}$$

We distinguish parameters in (i) potentially evolvable traits, denoted by the letter u and part of the trait vector $\mathbf{u}$; (ii) other non-evolvable parameters involved in the cognitive functions or the individuals' life cycle, for which the evolutionary dynamics would be constrained or trivial, are denoted

by λ, if affecting the time component of learning, or ρ, if affecting the intensity component.

As-actor circuit

The as-actor response is common to all individuals independently of their strategy. The probability of an appropriate response B is:

$$p_B(x, t, \mathbf{u}) = \Pr(a_{+i} \,|\, s_{+i};\; x_+, t_+) \tag{8}$$

$$= \Pr(a_{+i} \,|\, \hat{a}_{+i};\; x_+, t_+)\Pr(\hat{a}_{+i} \,|\, \hat{s}_{+i};\; x_+, t_+)\Pr(\hat{s}_{+i}, s_{+i}) \tag{9}$$

$$= \gamma_+ \, l(x_+, t_+)\, \alpha(x_+, u_B), \tag{10}$$

where t^+, the learning time for as-actor stimuli, would depend on the specific learning algorithm considered. Here, we want to provide a general treatment, and since we only care about the rate of learning, we only distinguish two classes of learning algorithms on the basis of their dependence on time. In the first class, each exposure to a stimulus improves future responses as $t^+ = p_+ t$, whether or not the focal individual performs an action. We define this mechanism as 'observational learning'. In the second class, that could be exemplified by any form of trial-and-error learning process, an individual improves future reactions on the basis of the outcome of previous responses. In this case, a learning event occurs only when the individual actually attempts a response. Hence, t^+ depends on an individual's own strategy, affecting the probability of an actual response:

$$t_+ = t\, p_+\, E_{x_+}[\alpha(x_+, u_B)] = t\, p_+ \int_0^\infty \alpha(x_+, u_B) \Pr(x_+)\, dx_+ \qquad , \tag{11}$$

where $\Pr(x_+)$ indicates the distribution of direct stimuli intensities x_+.

Finally, we have an explicit expression for P_B, the main component of the as-actor fitness:

$$P_B = E_{x_+, t_+}[p_B] = E_{x_+, t_+}[\; \alpha(x_+, u_B)\, l(x_+, t_+)\;]$$

$$= \int_0^\infty \int_0^\infty \alpha(x_+, u_B)\, l(x_+, t_+)\, \Pr(x_+)\, \Pr(t)\, dt\, dx_+\, . \tag{12}$$

As-observer circuit and strategy types

When an individual acts as an observer, the highest fitness payoff is attained by construction for response D. Hence all mind-reading strategies are expected to evolve maximizing P_D, the expectation of an appropriate social response.

Fixed Response strategies - F

The mapping providing the highest average payoff is the one matching the most common environmental state e_{max}. Therefore the average payoffs is proportional to the fraction of time spent in e_{max}, $\Pr(e_{\max})$:

$$\pi_-^F = P_D^F\, d^- = E[p_B^\circ\, p_D^F]\, d^- . \tag{13}$$

where

$$p_D^F = \Pr(e_{\max})\, \alpha^\bullet(x_-, u_B). \tag{14}$$

For environments changing at a constant rate we have:

$$P_D^F = \frac{\lambda_d}{\lambda_d + \lambda_e}\, E[p_B^\circ\, \alpha^\bullet(x_-, u_B)]\, . \tag{15}$$

Associative strategies - P

Schematically we can represent in a similar way the as-actor and P-strategies as-observer circuits:

$$\begin{aligned} &\text{as-actor circuit} && S^+ \to \hat{S}^+ \xrightarrow{l(x^+,t_+)} \hat{A}^+ \xrightarrow{\alpha(x^+,u_{\alpha+})} A^+ \\ &\text{as-observer P strategy} && S^- \to \hat{S}^- \xrightarrow{l(x^-,t_-)} \hat{A}^- \xrightarrow{\alpha(x^-,u_{\alpha-})} A^- \end{aligned} \tag{16}$$

By assuming null payoffs for 0 responses, the fitness component for a P observer is simply $\pi_-^P = P_D^P\, d^-$. For a focal P individual, P_D^P is equal to

$$P_D^P = E[p_B^\circ\, p_D^P], \qquad p_D^P(x_-, t_-, \mathbf{u}) = \alpha^\bullet(x_-, u_B) l^\bullet(x_-, t_-)\, . \tag{17}$$

Note that whereas for a focal actor the learning time t_+ depends only on its own strategy, for an observer it is also necessary that the observed actor's behavior is informative. Hence, t_- depends on p_- and p_B°. In the cases of observational and trial-and-error learning we have respectively:

$$t_- = p_-\, E[p_B^\circ]\, t\ , \tag{18}$$

$$t_- = p_-\, E[\alpha^\bullet(x_-, u_B)\, p_B^\circ]\, t\ . \tag{19}$$

We develop here the former case as:

$$t_- = E_{\mathbf{u}^\circ}\left[p_- \int_0^\infty \int_0^\infty \int_0^\infty l^\circ(x_+, t+) \alpha^\circ(x_-, u_B) \Pr(x_+, x_-) \Pr(t_+)\, dx_+\, dx_-\, dt_+\right] t. \tag{20}$$

Simulative strategies - S

In order to represent S we introduce a new class of cognitive functions, *simulative functions*. These functions allow an observer to map a social cue into an as-actor stimulus representation. Biologically, it has been suggested that mirror neurons, possibly developed through simple associative learning, might serve this purpose. For more complex or emotionally relevant stimuli, structures like the anterior insula and anterior medial cingulate cortex have been suggested [1]. Here, we only focus on the efficiency of this process, i.e. the probability of its success, and its possible role in the modulation of empathy and the responses of simulative strategies, i.e. the effect on the intensity of neural representations.

To this aim, we represent simulation as a two step process: in the first, social stimuli are mapped to neural representation of the corresponding stimuli as-if perceived by an actor; in the second, the inferred as-actor response is used by the observer to choose a social response. Formally, we define these *simulation functions* as any function mapping an actions or stimuli representations within the same space, either $\hat{S}$ or $\hat{A}$, but different subspaces. In particular we refer to the subspaces of as-actor (i.e. $\hat{S}^+, \hat{A}^+$) and as-observer representations (i.e. $\hat{S}^-, \hat{A}^-$). Thus, we can distinguish the two simulation functions as:

- $\gamma_s : \hat{S}^- \to \hat{S}^+$, maps a social stimulus representation $\hat{s} = (s_{-i}, x) \in \hat{S}^-$ to an as-actor one $\hat{s} = (s_{+i}, y) \in \hat{S}^+$, allowing for the representation of a social stimulus "as if" it was directly perceived as an actor. Thus:

$$\gamma_s| \quad \hat{s} = (s_{-i}, x) \in \hat{S}^- \to \hat{s} = (s_{+i}, u_D\, x) \in \hat{S}^+ \tag{21}$$

- $\gamma_a : \hat{A}^+ \to \hat{A}^-$, operates similarly but in a reverse fashion, so that $\hat{a} = (a_{+i}, x) \in \hat{A}^+$ is mapped to $\hat{a} = (a_{-i}, y) \in \hat{A}^-$. Therefore, the representation of an inferred as-actor action can be used to select the appropriate social response and produce the corresponding neural configuration:

$$\gamma_a| \quad \hat{a} = (a_{+i}, u_D\, x) \in \hat{A}^+ \to \hat{a} = (a_{-i}, x) \in \hat{A}^+ \quad . \tag{22}$$

The possibility that during the simulation process, as-actor representations are modulated in intensity, is implemented as an evolvable trait u_D, described later in details. First, we describe the flow of a simulative circuit:

1. An observer perceives a social cue s_{-i} as a representation $\hat{s}_{-i} \in \hat{S}^-$ (Fig.1c,d, first continuous arrow).
2. Instead of using social experience, a simulative strategy maps the social cue representation to

its correspondent as-actor representation, $\hat{s}_{+i} \in \hat{S}^{+}$, via γ_s (Fig.1c-d, first dashed arrow).

3. The observer uses its own as-actor circuit in order to infer an appropriate response to $\hat{s}_{+i}$, namely $\hat{a}_{+i}$ (Fig.1c-d, gray arrow). This representation corresponds to an inference about the actor's behavior. The core of the as-actor circuit is the learning function l, mapping $\hat{s}_{+i}$ to $\hat{a}_{+i}$ on the basis of the as-actor experience t_{+}.

4. Finally, the inference $\hat{a}_{+i}$ can be mapped to an appropriate social response, $\hat{a}_{-i}$, via γ_a (Fig.1c-d, second dashed arrow). Hence, γ_s and γ_a describe in the model the efficiency of the simulation process, and the possible loss of information associated to it.

We already suggested that a potential advantage of S is to take advantage of private information acquired as an actor. In order to gain access to this information however, a simulative strategy has to activate, at least partially, the corresponding as-actor circuits ($\hat{s}_{+i}, \hat{a}_{+i}$, Fig.1). This represents an inherent major constraint of such strategies. In fact, simulated action representations are part of the response machinery to direct stimuli. Therefore, an activation of these simulated neural configurations, if comparable to cases of a direct perception of the stimulus, might trigger the downstream responses. For an observer, this leads to the activation of as-actor responses. This process, also called facilitation, might interfere with an appropriate social response, and in our model it can be associated with a cost. Conservatively, we assumed that whenever an action representation activates the downstream action, the latter hinders a further response. Therefore, a C response occurs when a simulated action representation $\hat{a}_{+i} \in \hat{A}^{+}$ results in the activation of a_{+i}. The probability $\Pr(a_{+i}|\hat{a}_{+i}) = \alpha(u_D\, x_{-}, u_B)$ is simply given by the activation function. Because of the risk of coordination, inhibitory processes of C are likely subjected to evolutionary constraints. Thus, in order to study their evolution, we investigated the evolutionary dynamics of key traits involved in this modulation. In our model, inhibition of coordination can be achieved in different ways:

- By adopting non-coordinating strategies like P or F. A variation of this mechanism is *temporal inhibition*, using social information whenever this is sufficient to infer actors' responses without adopting a simulative strategy. This is explored in Section 2.4 as a continuous trait u_S.

- By evolving the as-actor circuit in order to increase its specificity and avoid activation in response to simulated action representations. This mechanism corresponds to structural changes of the as-actor circuit, thus influencing the behavior of an organism even as an actor. Since B responses as an actor are affected, we represent this trait as an evolvable trait denoted as u_B.

- By modulating the way the as-actor network is recruited during simulation. We model this with a continuous trait u_D.

Using the last two mechanisms, an organism can inhibit coordination while still adopting a simulative strategy. We model these mechanisms by exploring the evolution of traits influencing the shape of the activation function, $\alpha(u_D\, x_-, u_B)$, which determines the probability of C responses. In this framework, these two alternative ways to avoid activation can be visualized as an increase of the threshold of α, u_B, or a decrease of the input intensity of the simulated neural configurations by reducing u_D.

For a focal S individual, the probabilities of C and D responses and the as-observer component of the fitness are:

$$p_C^S = \gamma_s^\bullet\, l^\bullet(u_D\, x_-, t_+)\, \alpha^\bullet(u_D\, x_-, u_B) \tag{23}$$

$$p_D^S = \gamma_s^\bullet\, l^\bullet(u_D\, x_-, t_+)\, \big(1 - \alpha^\bullet(u_D\, x_-, u_B)\big)\gamma_a^\bullet\, \alpha^\bullet(x_-, u_B) \tag{24}$$

$$\begin{aligned}\pi_-^S &= P_D^S\, d^- - P_C^S\, c^- \\ &= E\big[p_B^\circ\, \big(p_D^S\, d^- - p_C^S\, c^-\big)\big].\end{aligned} \tag{25}$$

Note that S strategies rely on as-actor information rather than on as-observer information, differently from P strategies. Thus, in this model, S strategies are entirely independent of t_-, and acquire information as t_+. We relax this assumption in Section 4, exploring a model in which both as-actor and as-observer information are used in simulation functions.

Section 2

Evolutionary analysis

Evolution of strategy types and u_D

In the frequency-independent case the evolutionary equilibrium value u_D^* is the global maximum of $\pi_-^S(u_D)$. Thus u_D^* is also an evolutionarily stable strategy (ESS), and satisfies:

$$\left.\frac{\partial P_D^S(u_D)}{\partial u_D}\right|_{u_D=u_D^*} d^- - \left.\frac{\partial P_C^S(u_D)}{\partial u_D}\right|_{u_D=u_D^*} c^- = 0 \tag{26}$$

and

$$\left.\frac{\partial^2 P_D^S(u_D)}{\partial^2 u_D}\right|_{u_D=u_D^*} d^- - \left.\frac{\partial^2 P_C^S(u_D)}{\partial^2 u_D}\right|_{u_D=u_D^*} c^- < 0. \tag{27}$$

The first condition indicates that u_D^* is a *singular point*, where the selection gradient $\frac{d\pi_-^S(u_D^*)}{du_D}$ is zero[2]. The second derivative condition guarantees the non-invasibility by small mutants and the presence of a fitness maximum. We denote the equilibrium strategy S characterized by u_D^* as S^*. Since π_-^S depends only on the focal u_D, for a singular strategy the ESS second derivative condition coincides with

the requirements of convergence stability (CS)[3, 4], namely S^* invades and dominates over all other S strategies. Therefore, we consider S^* for all analyses contrasting different types of mind-reading strategies.

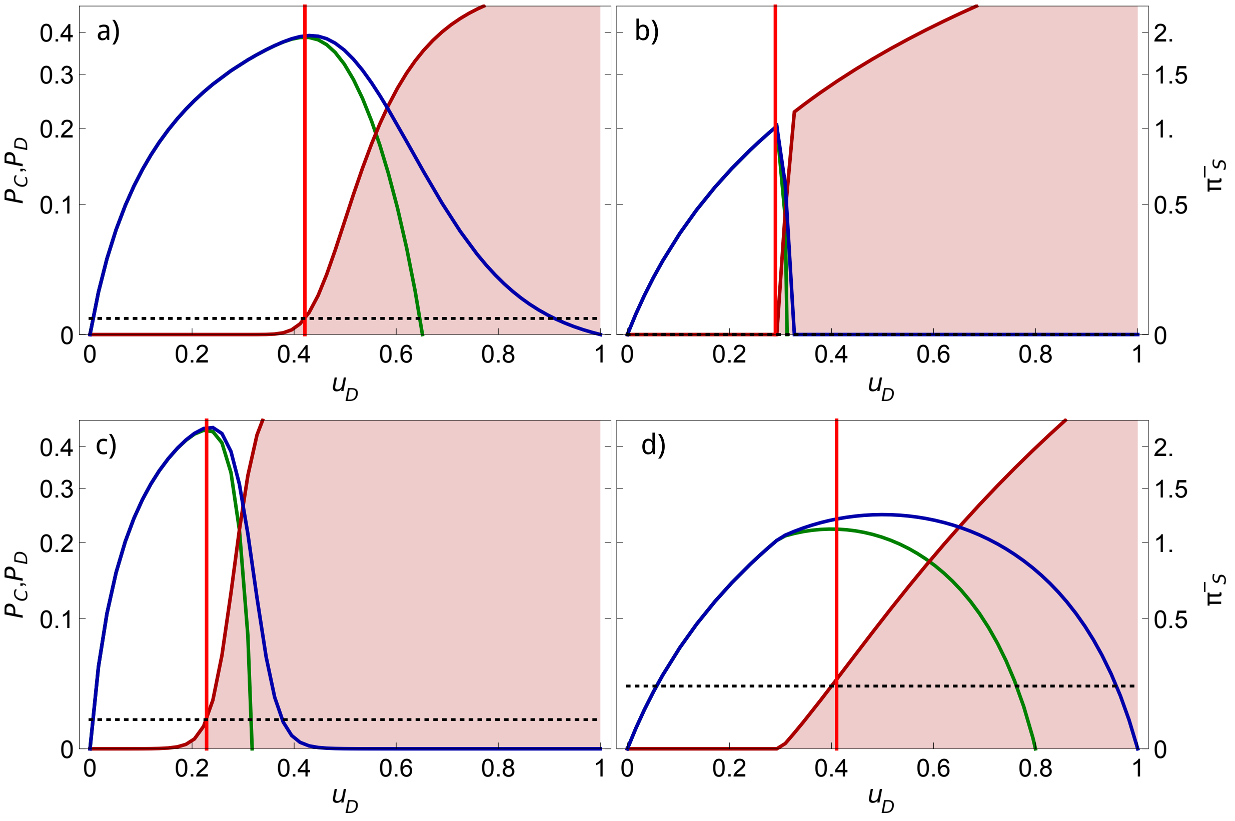

Figure S 1: P_C^S (dark red), P_C^S (dark blue) and π_-^S (green) for different values of u_D (x-axis) for different models of inhibition. Respectively, activation functions are represented as a step (a), sigmoid (b) or step-linear functions (d) and (c). In fig.(a), intensities are normally distributed with $x \sim N(0.5, 0.1)$. For all the other figures ($x = 1$). The parameters considered are $u_B = 0.3$, $\lambda_l = 1$, $\rho_x = 1$, $\gamma_s = 1$, $\gamma_a = 1$, $d^- = d^+ = 5$, $c^- = 2$. ρ_a is considered equal to $13.8155/u_B$ for (a),(b) and (c). In (d) $\rho_a = 1/(1 - u_B) = 1.428$.

Equation 26 shows that the evolution of simulative strategies is subjected to a trade-off, regardless of the specific mathematical functions used to represent the cognitive processes: the as-actor network will be recruited more (higher u_D) if this increases the accuracy of social inferences (higher P_D^S); however, if this results in an increased risk of coordination, the as-actor network will be recruited less (lower u_D, and in turn lower P_C^S). An at least partial inhibition occurs whenever the intensities of simulated stimuli representations $E[u_D\, x_-]$ are on average lower than the intensities of stimuli perceived as an actor $E[x_+]$. When $u_D^* \to 0$ simulative strategies completely inhibit coordination.

We are interested in determining under which conditions the non-trival case in which an internal equilibrium exists and the as-actor network is partially recruited, while coordination still occurs, i.e. $u_D^* > 0, P_C^S > 0$. We start by exploring the case of cheap coordination ($c^- = 0$), for which only P_D^S

affects the fitness of an S observer, and eq.26-27 simplify into:

$$\left.\frac{dP_D^S(u_D)}{du_D}\right|_{u_D=u_D^*} = 0\,, \left.\frac{d^2P_D^S(u_D)}{du_D^2}\right|_{u_D=u_D^*} < 0. \tag{28}$$

Recalling eq.23-24, when considering the simplified model in which $x_+ = x_- = 1$:

$$\left.\frac{d\,l^\bullet(u_D,t_+)}{du_D}\right|_{u_D=u_D^*} = \left.\frac{d\,\alpha^\bullet(u_D,u_B)\,l^\bullet(u_D,t_+)}{du_D}\right|_{u_D=u_D^*} \tag{29}$$

$$\left.\frac{d^2\,l^\bullet(u_D,t_+)}{d^2u_D}\right|_{u_D=u_D^*} < \left.\frac{d^2\,\alpha^\bullet(u_D,u_B)l^\bullet(u_D,t_+)]}{d^2u_D}\right|_{u_D=u_D^*}. \tag{30}$$

The selection gradient is positive and u_D evolves to higher values when:

$$\frac{1}{l^\bullet(u_D,t_+)}\frac{d\,l^\bullet(u_D,t_+)}{du_D} > \frac{1}{1-\alpha^\bullet(u_D,u_B)}\frac{d\,\alpha^\bullet(u_D,u_B)}{d\,u_D} \tag{31}$$

This equation exemplifies several points. First, a benefit in terms of more efficient mind-reading is necessary for u_D to evolve to non-zero values and for the existence of a singular point with non-zero coordination, i.e. P_D^S must be an increasing function of u_D. Second, u_D increases as long as the relative improvement in social inferences is larger than the relative loss of appropriate responses due to coordination. For values of $u_D \approx 0$ coordination is mostly inhibited, thus u_D evolves to higher values. However, as u_D increases, $\frac{1}{l^\bullet(u_D,t_+)}$ decreases and $\frac{1}{1-\alpha^\bullet(u_D,u_B)}$ increases. Thus, u_D can evolve to a singular point in which inhibition occurs even in absence of explicit costs for coordination, just because coordination will interfere with the appropriate social response D.

Note that typically, activation/inhibitory functions are highly convex: on a single variable, the discriminatory ability of such functions is determined by their slope, and their steep change allows for the classification of non-relevant and relevant inputs/stimuli. In biologically-based models of neurons and brain activation, as well as in computational modeling, the typical activation function is a sigmoid function: a steep threshold ensures the discrimination of low-intensity/below-threshould stimuli, that are inhibited, from high-intensity relevant ones (see [5, 6, 7, 8, 9, 10] or [11, 12, 13] for comprehensive reviews on the topic, and [14] for an application in mirror systems). Thus, the evolution of u_D is strongly constrained by the threshold of the activation function: inhibition becomes inefficient if u_D increases beyond the activation threshold and generally cannot evolve to higher values. Thus a singular point u_D^* generally occurs for any typical activation function/inhibitory mechanism. Note that also the second ESS condition is easily satisfied for any smooth activation/inhibition function. In terms of the u_D parameter space, this ensures the presence of a low u_D region where D prevails over C, and the presence of a high u_D region, where coordination strongly hinder appropriate responses. When

α is smooth enough (i.e. differentiable), u_D always partially "climbs" the activation function to an internal equilibrium value u_D^* that is independent of the payoffs, and depends only on the shape of the cognitive functions. Note from eq.28 that in the most general formulation of the model, what is required for an internal equilibrium is that the expectation of P_D^S is continuous. This condition can be in principle fulfilled even for non-continuous activation functions, representing extremely discriminative forms of inhibition: for example, any stochasticity in the stimuli intensity (i.e. their intensity is not always identical) would lead to continuous expectations even for non-continuous activation functions. An example is a step activation function, with distributed normally stimuli intensities, as shown in Fig.S1, and simulated in Section 5.

Similar conclusions can be drawn in the more general case of costly coordination ($c^- > 0$). When both l and α are increasing functions of u_D, u_D evolves towards higher values as long as

$$\frac{d\,P_D^S}{d\,u_D} / \frac{d\,P_C^S}{d\,u_D} \geq \frac{c^-}{d^-} \quad , \tag{32}$$

where equality holds for the singular strategy u_D^*. In terms of the cognitive functions, eq.26-27 can be rewritten as:

$$\frac{\partial E[p_B^{\circ} l^{\bullet}(u_D\, x_-, t_+)\, \alpha^{\bullet}(x_-, u_B)\gamma_a^{\bullet}\, d^-]}{\partial u_D} = \frac{\partial E[p_B^{\circ} l^{\bullet}(u_D\, x_-, t_+)\, \alpha^{\bullet}(u_D\, x_-, u_B)\big(\alpha^{\bullet}(x_-, u_B)\gamma_a^{\bullet}\, d^- + c^-\big)]}{\partial u_D}, \tag{33}$$

$$\frac{\partial^2 E[p_B^{\circ} l^{\bullet}(u_D\, x_-, t_+)\, \alpha^{\bullet}(x_-, u_B)\gamma_a^{\bullet}\, d^-]}{\partial^2 u_D} < \frac{\partial^2 E[p_B^{\circ} l^{\bullet}(u_D\, x_-, t_+)\, \alpha^{\bullet}(u_D\, x_-, u_B)\big(\alpha^{\bullet}(x_-, u_B)\gamma_a^{\bullet}\, d^- + c^-\big)]}{\partial^2 u_D}, \tag{34}$$

where we remind that $p_B^{\circ} = \alpha^{\circ}(x_+, u_B) l(x_+, t_+)$. Whereas in the cheap coordination case, u_D^* is independent of the payoffs, for costly coordination the explicit cost c^- adds up to the intrinsic cost of C responses. Expectedly, a higher c^- reduces the equilibrium value u_D^*.

For readability, we recall our previous simplifying assumptions. Hence, we consider again stimuli intensities equal to 1 and in turn simulated stimuli with intensities u_D. We also assume that non simulated representations are not inhibited i.e. $\alpha(1, u_B) = 1$. In this case eq.33-34 equal:

$$\frac{\partial E[l^{\bullet}(u_D, t_+)]}{\partial u_D}\, \gamma_a^{\bullet}\, d^- = \frac{\partial E[l^{\bullet}(u_D, t_+)\, \alpha^{\bullet}(u_D, u_B)]}{\partial u_D}(\gamma_a^{\bullet}\, d^- + c^-), \tag{35}$$

$$\frac{\partial^2 E[l^{\bullet}(u_D, t_+)]}{\partial^2 u_D}\, \gamma_a^{\bullet}\, d^- < \frac{\partial^2 E[l^{\bullet}(u_D, t_+)\, \alpha^{\bullet}(u_D, u_B)]}{\partial^2 u_D}(\gamma_a^{\bullet}\, d^- + c^-), \tag{36}$$

Again, both conditions are satisfied when l and α are increasing functions of u_D, and α is strongly convex.

We explore numerically the evolution of u_D for simulative strategies in Fig S1. As we already

mentioned, when P_C increases smoothly with u_D, u_D^* is a Continuously Stable Strategy (CSS), as shown in the pairwise invasibility plot (PIP) (Fig.S2a)[4]. S^* invades all other simulative strategies regardless of the level of environmental complexity, since the sign of the invasion fitness remains the same regardless of the parameters describing environmental variation. Thus, the same PIP and the same value u_D^* hold for any value of λ_e or p_-. As a consequence, $\alpha(u_D^*, u_B)$, and in turn the ratio between P_C and P_D, remain constant, despite the fact that both expectedly decrease with λ_e and with a reduction in learning time (Fig.S2b-c).

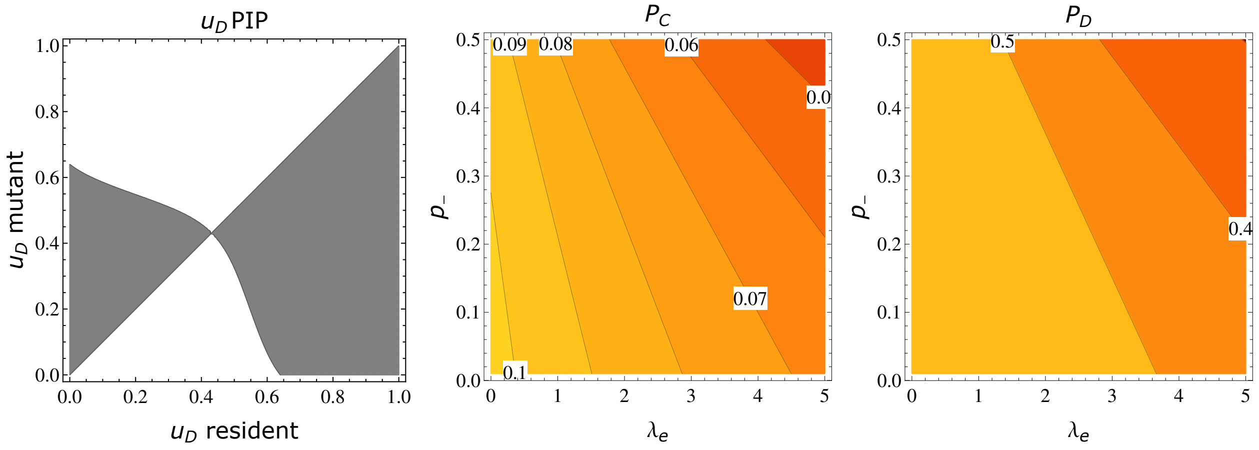

Figure S 2: (a) Pairwise Invasibility Plot (PIP) for the evolution of u_D. The values of u_D for the resident population and the mutant are reported respectively on the x and y axis. Gray regions indicate a higher fitness for the mutant, while white ones higher fitness for the resident population. u_D^* is an ESS, as we can see from the fact both higher or lower mutant u_D values along a vertical line passing through u_D^* would confer a lower fitness compared to the resident. u_D^* is also CS, since mutant with slightly higher u_D values than the resident population invade when below u_D^* (gray above the diagonal), and the pattern is reversed for values larger than u_D^*. Mutant S^* invades all other simulative strategies, since the horizontal line crossing u_D^* lies entirely in gray regions. (b-c) Contour plots for P_C (b) and P_D (c) for different values of λ_e (x-axis) and p_- (y-axis). Darker shades indicate smaller probability values, indicated by the labels reported in the plot. For all the figures u_D^* equals 0.4313, and it is obtained for the following parameters: $x = 1, u_B = 0.6, \rho_a = 13.8155/u_B, \rho_x = 5, \lambda_l = 20, \gamma_a = 1, d^- = d^+ = 5, c^- = c^+ = 3$, assuming a sigmoid activation function and a step linear l_x.

Evolution of strategy types with different u_B

Competition between strategy types

We now consider the evolution of more radical forms of inhibition of C events, acting through a structural modification of the as-actor network itself, and thus affecting also the behavior of an individual as an actor. We model this by exploring the evolution of a continuous trait u_B. For P and F, the evolutionary dynamics are trivial: since coordination never occurs, inhibition is always deleterious, and u_B converges to the minimum possible value. We define this equilibrium value as u_B^{FP}. Regarding S strategies, u_B affects in opposite direction two processes: higher u_B values determine a more

efficient inhibition of coordination, increasing the ratio between P_D and P_C; however higher u_B values determine a higher intensity threshold also when the focal individual is an actor, reducing P_B.

We perform a general stability analysis of the competition between the three different strategy types, investigating cases for which $u_B^S > u_B^{FP}$. We can rewrite the replicator systems as:

$$
\begin{aligned}
\frac{dy^F}{dt} &= \quad y^F \left(\pi^F(u_B^{FP}, u_D, u_B^\circ) - \phi(u_B^\circ, u_D)\right) \\
\frac{dy^P}{dt} &= \quad y^P \left(\pi^P(u_B^{FP}, u_D, u_B^\circ) - \phi(u_B^\circ, u_D)\right) \\
\frac{dy^S}{dt} &= \quad y^S \left(\pi^S(u_B^{S}, u_D, u_B^\circ) - \phi(u_B^\circ, u_D)\right),
\end{aligned} \tag{37}
$$

where we expressed all fitness functions in terms of the evolvable traits. For simplicity, we focus first on the competition between different types of strategy, neglecting the evolution of continuous traits. This corresponds to assuming a separation in time scale [4, 2] between the evolution of strategic types, and the evolutionary fine-tuning of continuous traits shaping the different cognitive functions. In section 2.4 we relax this assumption by introducing a different framework. When the continuous traits are

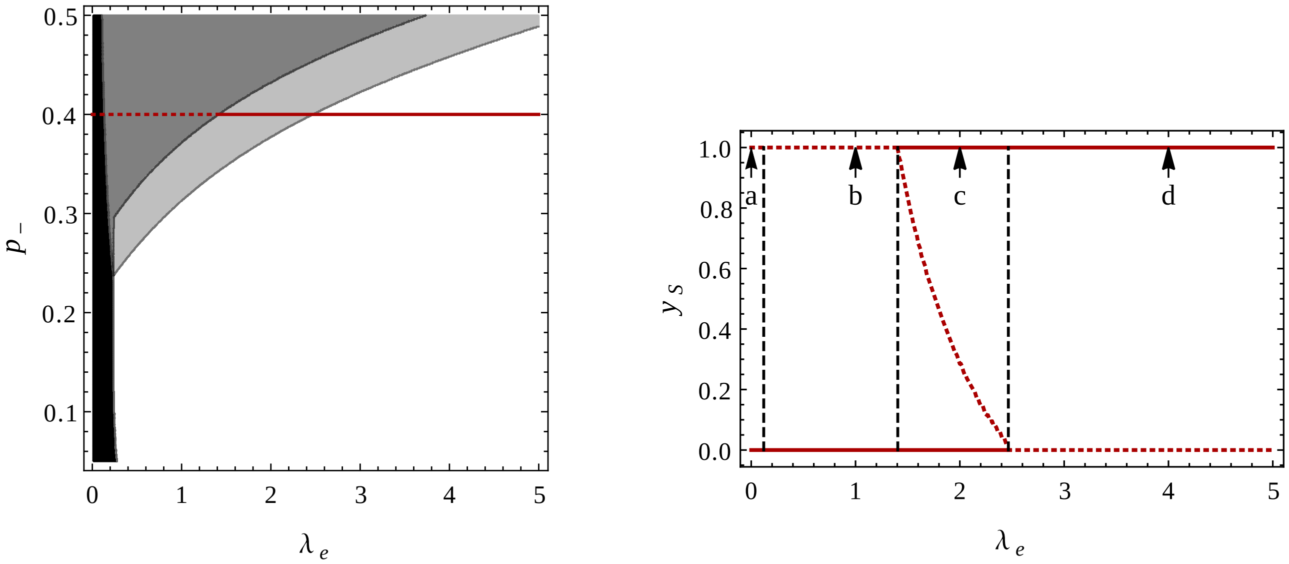

Figure S 3: Left) S (white), P (dark grey) and F (black) invasion domains when u_B^S differs from u_B^{FP} (here $u_B^S = 0.3$ and $u_B^{FP} = 0.05$). Bistable regions are shown in light gray. The red line indicates the p_- value for which the bifurcation plot shown in the right panel is obtained. We considered different values of λ_e (x-axis) and p_- (y-axis) and identically distributed unitary intensities ($x = 1$). The parameters considered are $u_B = 0.3$, $\lambda_l = 1$, $\rho_x = 1$, $\gamma_s = 1$, $\gamma_a = 1$, $d^- = d^+ = 5$, $c^- = c^+ = 2, \rho_a = 13.8155/u_B$ for a sigmoid activation function. Right) Bifurcation plot for the parameters indicated in the left panel. The critical points for the frequency of simulative strategies are shown as red lines, continuous when stable and dotted when unstable. Dotted vertical lines indicate transitions between invasion domains. The arrows indicate values for which the ternary plots in Fig.S4 are obtained.

fixed, different values of u_B for simulative and non simulative strategies lead to frequency-dependent

dynamics. The overall pattern in the competition between the different strategies is similar to the frequency independent case: S dominates for more variable environments, whereas F dominates for more stable ones (Fig.S3). However, bistability can appear at the transition between the domain of attractions of S and P: the initial frequency of the two strategies determines which one dominates. This occurs when S strategies have higher u_B than P or F strategies. When actors have lower u_B less B responses occur, mimicking the effects of increased environmental variation on learning: the effective learning times t_+ and t_- are reduced. Hence, higher frequencies of S strategies stabilize the S-only equilibrium, and vice versa.

Evolution of u_B

We investigate here which specific values of u_B the population would evolve to, when simulative strategies are present. We adopt the adaptive dynamics assumptions of small and rare mutations and of a population at the ecological equilibrium. Successive mutants invade in the direction provided by

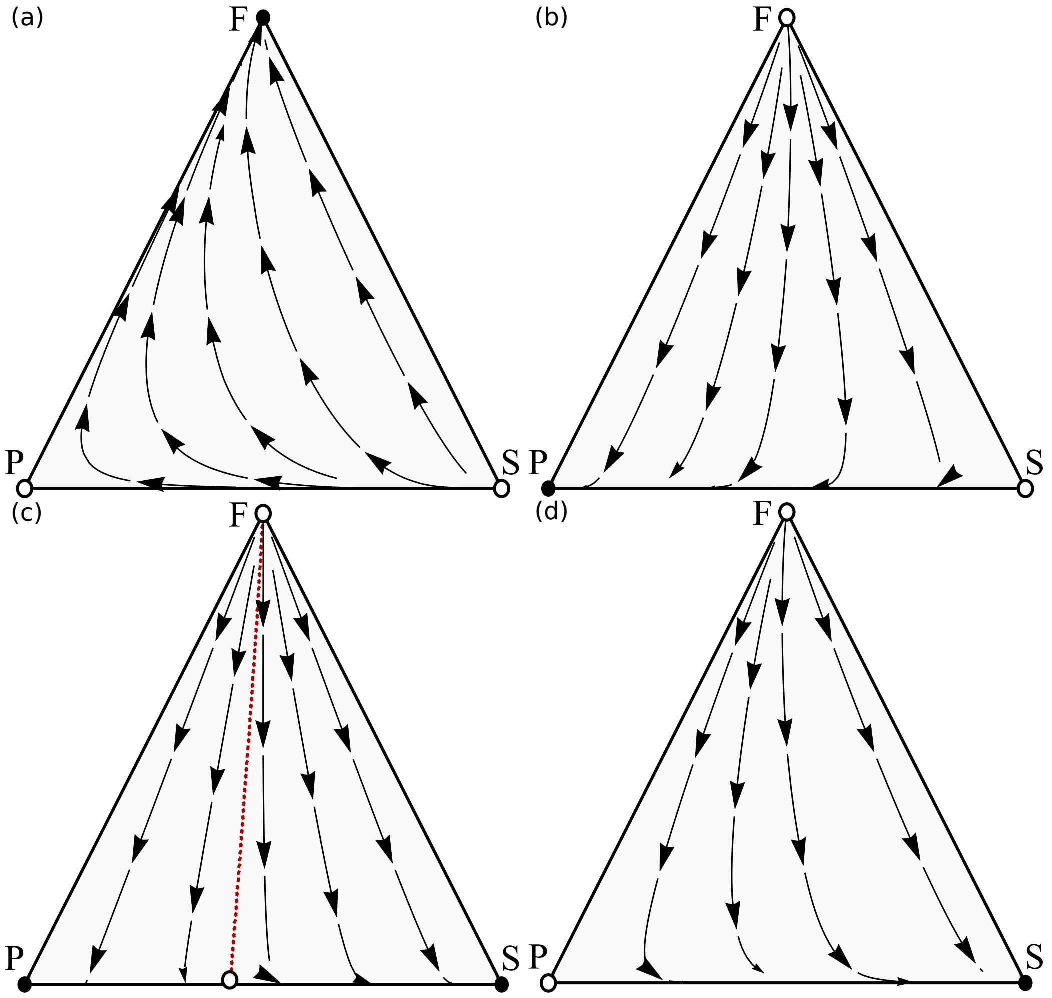

Figure S 4: Ternary plots for the combination of parameters indicated in Fig.S3. Black dots indicate stable equilibria, white ones repellors. Red dotted lines separate invasion domains.

the selection gradient, expressed below for a focal mutant $u_B^\bullet$, in resident population with trait u_B°:

$$\frac{d(\pi^S(u_B^\bullet,u_B^\circ)-\phi(u_B^\circ))}{du_B^\bullet}\bigg|_{u_B^\bullet=u_B^\circ} = \frac{\partial\pi^S(u_B^\bullet,u_B^\circ)}{\partial u_B^\bullet}|_{u_B^\bullet=u_B^\circ} \tag{38}$$

$$= (p_+-p_-)\frac{\partial\pi_+(u_B^\bullet)}{\partial u_B^\bullet}|_{u_B^\bullet=u_B^\circ} + p_-\frac{\partial\pi_\pm(u_B^\bullet,u_B^\circ)}{\partial u_B^\bullet}|_{u_B^\bullet=u_B^\circ} + p_-\frac{\partial\pi_-(u_B^\bullet)}{\partial u_B^\bullet}|_{u_B^\bullet=u_B^\circ}\,.$$

We can develop the selection gradient for the as-actor (including the as-lone actor and as-observed actor terms) and observer components in terms of the cognitive functions, respectively:

$$(p_+-p_-)\frac{\partial\pi_+(u_B^\bullet)}{\partial u_B^\bullet}|_{u_B^\bullet=u_B^\circ} + p_-\frac{\partial\pi_\pm(u_B^\bullet,u_B^\circ)}{\partial u_B^\bullet}|_{u_B^\bullet=u_B^\circ} = \tag{39}$$

$$\frac{\partial}{\partial u_B^\bullet}|_{u_B^\bullet=u_B^\circ}\,E[p_B(u_B^\bullet)\big(p_+\,b+\,p_-(-p_D(u_D^\circ,u_B^\circ)d^+ + p_C(u_D^\circ,u_B^\circ)c^+)\big)]\,,$$

$$p_-\frac{\partial\pi_-(u_B^\bullet)}{\partial u_B^\bullet}|_{u_B^\bullet=u_B^\circ} = \tag{40}$$

$$\frac{\partial}{\partial u_B^\bullet}|_{u_B^\bullet=u_B^\circ}\,E[p_B(u_B^\circ)\big(p_+\,b\,+p_-(p_D(u_D,u_B^\bullet)d^- - p_C(u_D,u_B^\bullet)c^-)\big)]\,.$$

The as-lone actor selection gradient (eq.39) is always negative for non-negative values of u_B, since higher u_B determines lower values of p_B. Note that the as-observed actor selection gradient can be either positive or negative, when it is deleterious to be observed and actors are very efficient, i.e. for the resident population $P_D\,d^+ > P_C\,c^+$ and $P_B b < P_D\,d^- - P_C\,c^-$. In this case any action is disadvantageous for an actor, because of the excessively costly interaction with a potential observer. Thus, a trivial equilibrium can exist if the as-actor selection gradient is negative, in which no action is performed by actors.

We are however interested in cases in which actors perform B responses that can be exploited by observers, and the as-actor selection gradient is negative. We already observed how for S observers, u_D evolves so to inhibit as-actor circuits, by achieving that simulated representations can be distinguished by stimuli perceived as an actor, i.e. $u_D\,E[x_-] < E[x_+]$. For u_B, contrary to the as-actor selection gradient, the as-observer selection gradient can be positive: when u_B is very low, i.e. $u_B << E[u_D\,x_-]$, inhibition never occurs. As u_B increases and approaches $E[u_D\,x_-]$, coordination gets inhibited, while responses B as an actor can still occur. When u_B is higher than $E[x_+]$ actions as an actor are inhibited. Therefore u_B generally evolves towards a singular point allowing the discrimination of simulated and direct stimuli.

We can obtain explicit expressions for u_B^* by assuming the same cognitive functions used in the

simplified step-linear model of the main text, with $0 \leq u_D \leq 1$. In this case the selection gradient is:

$$\left.\frac{d\pi^S(u_B^\bullet, u_B^\circ)}{du_B^\bullet}\right|_{u_D^\bullet = u_D^\circ} = \begin{cases} 0 & : u_B + 1/\rho_a \leq u_D \\ (d^- + c^-)p_-\, u_D\, \rho_a & : u_B < u_D < u_B + 1/\rho_a \leq 1 \\ -b(1-p_-)\rho_a + (d^- + c^-)p_-\, u_D\, \rho_a^2(1-u_B) & : u_B < u_D \leq 1 < u_B + 1/\rho_a \\ -b(1-p_-)\rho_a & : u_D < u_B \leq 1 < u_B + 1/\rho_a \end{cases} \tag{41}$$

As we can see in eq.41, as long as inhibition does not hinder as-actor responses ($u_B << 1$), the as-actor fitness component is negligible and the selection gradient is non-negative. In particular, for steep activation functions, when $u_D \approx u_B$, the as-observer selection gradient strongly increases. Vice versa, when $u_B + 1/\rho_a$ approaches 1 and the as-actor behavior is compromised, the selection gradient changes sign.

Here we have to distinguish two different cases. When ρ_a is very high, hence α very discriminative, a full inhibition of coordination can be achieved, by evolving u_B to any values between u_D and $1 - 1/\rho_a$. However, if $1/\rho_a > 1 - u_D$, a full inhibition cannot be achieved without affecting the as-actor responses of the focal individual. Hence, a tradeoff between efficient inhibition of C events and deleterious inhibition of B responses emerge: u_B will evolve towards the singular strategy $u_B^* = 1 - (b(1-p_-))/(p_- r \rho_a(d^- + c^-))$. The value of u_B^* decreases with $b(1-p_-)/p_-$, reflecting the tradeoff between the as-actor and as-observer payoffs. A singular strategy u_B^* is Convergence Stable (CS) or an ESS respectively when:

$$\frac{d}{du_B^\circ}\left(\left.\frac{\partial\,\pi(u_B^\bullet, u_B^\circ)}{\partial\,u_B^\bullet}\right|_{u_B^\bullet = u_B^\circ}\right)\Bigg|_{u_B^\circ = u_B^*} < 0 \tag{42}$$

$$\left.\frac{\partial^2\,\pi(u_B^\bullet, u_B^\circ)}{\partial\,u_B^{\bullet 2}}\right|_{u_B^\bullet = u_B^\circ,\, u_B^\circ = u_B^*} \leq 0\,. \tag{43}$$

In this case, both conditions are always verified since:

$$\frac{d}{du_B^\circ}\left(\left.\frac{\partial\,\pi(u_B^\bullet, u_B^\circ)}{\partial\,u_B^\bullet}\right|_{u_B^\bullet = u_B^\circ}\right)\Bigg|_{u_B^\circ = u_B^*} = -(c^- + d^-)\,p^- u_D \rho_a^2 < 0 \tag{44}$$

$$\left.\frac{\partial^2\,\pi(u_B^\bullet, u_B^\circ)}{\partial\,u_B^{\bullet 2}}\right|_{u_B^\bullet = u_B^\circ,\, u_B^\circ = u_B^*} = 0\,. \tag{45}$$

Regarding coordination, a similar behavior occurs when u_D or u_B evolve, as a smooth α leads to the evolution of occasional coordination once an S is adopted. We investigate the stability conditions numerically, for a sigmoidal activation function (Pairwise Invasibility Plot in Fig.S5a). Again, we identify a CSS, with non-zero probability of coordination. However, smooth α also determine the presence of an internal repellor, hindering the evolution of u_B to higher values when the initial resident

trait is lower than the repellor. For a wide range of parameter, larger mutational steps still guarantee the convergence to the CSS. This can be seen in the PIP(Fig.S5a) for the horizontal line passing through the CSS (every mutant with that $u_B = u_B^*$ invades and converge to the CSS). The appearance of this repellor is due the fact that for cases different than the α-step-linear, nothing guarantees that the as-actor component of the fitness is necessarily smaller than the as-observer component when $u_B << u_D$. For a sigmoidal α, both the as-observer and as-actor component of the selection gradient approach 0, but the latter could be larger. When the benefit of as-observer responses becomes small compared to the as-actor component of the fitness (very low p_-, $a >> d^- + c^-$), a repellor appears, as shown in Fig.S5a. Furthermore, in some instances the as-actor fitness component always exceeds the as-observer one, the CSS disappears and only a repellor exists: at that point u_B converges to the minimum possible value, approaching u_B^{FP} (Fig.S5b). This occurs for extremely low value of p_- (in Fig.S5b $p_- = 0.01$) and high environmental complexity, leading to an abrupt decrease in u_B and P_D, and a local increase in coordination events (Fig.S5c-f). These results reveal a non-monotonic effect of environmental complexity and p_- on the evolution of simulative strategies: even though low p_- and high complexity favor S against other strategies, simulation collapses when p_- and λ_e increase excessively. The effects of decreasing p_- can be also observed on P_D and P_C, both increasing to finally decrease abruptly for extreme combinations of λ_e and low p_-.

Simultaneous evolution of u_B and u_D

These aspects can be further investigated by looking at the simultaneous evolution of u_D and u_B. In this case, a singular strategy is given by the combination of trait u_D^* and u_B^* such that the selection gradient for both trait is zero, hence:

$$D_{u_B}(u_B^\circ, u_D^\circ) = \left.\frac{\partial \pi^S(u_D^\circ, u_B^\bullet, u_B^\circ)}{\partial u_B^\bullet}\right|_{u_B^\bullet = u_B^\circ} = 0 \tag{46}$$

$$D_{u_D}(u_B^\circ, u_D^\circ) = \left.\frac{\partial \pi^S(u_D^\bullet, u_B^\circ, u_B^\circ)}{\partial u_D^\bullet}\right|_{u_D^\bullet = u_D^\circ} = \left.\frac{\partial \pi_-^S(u_D^\bullet, u_B^\circ, u_B^\circ)}{\partial u_D^\bullet}\right|_{u_D^\bullet = u_D^\circ} = 0 \tag{47}$$

The system is characterized by a global attractor S^* with u_D^* and u_B^*, as shown in Fig.S6 (representative of all the parameters tested). We have already shown that a CSS always exists for u_D, given any u_B. The same is not true for u_B, since for high u_D an internal repellor might lead u_B to evolve towards small values. However, the internal repellor, which is present when only u_B evolves, generally disappears when u_D and u_B evolve simultaneously, except for extreme values of b. This is due to the fact that u_D quickly evolves to lower values when $u_D > u_B$. Hence, even for low u_B, eventually $u_B \simeq u_D$, and the as-observer selection gradient for u_B sharply increases.

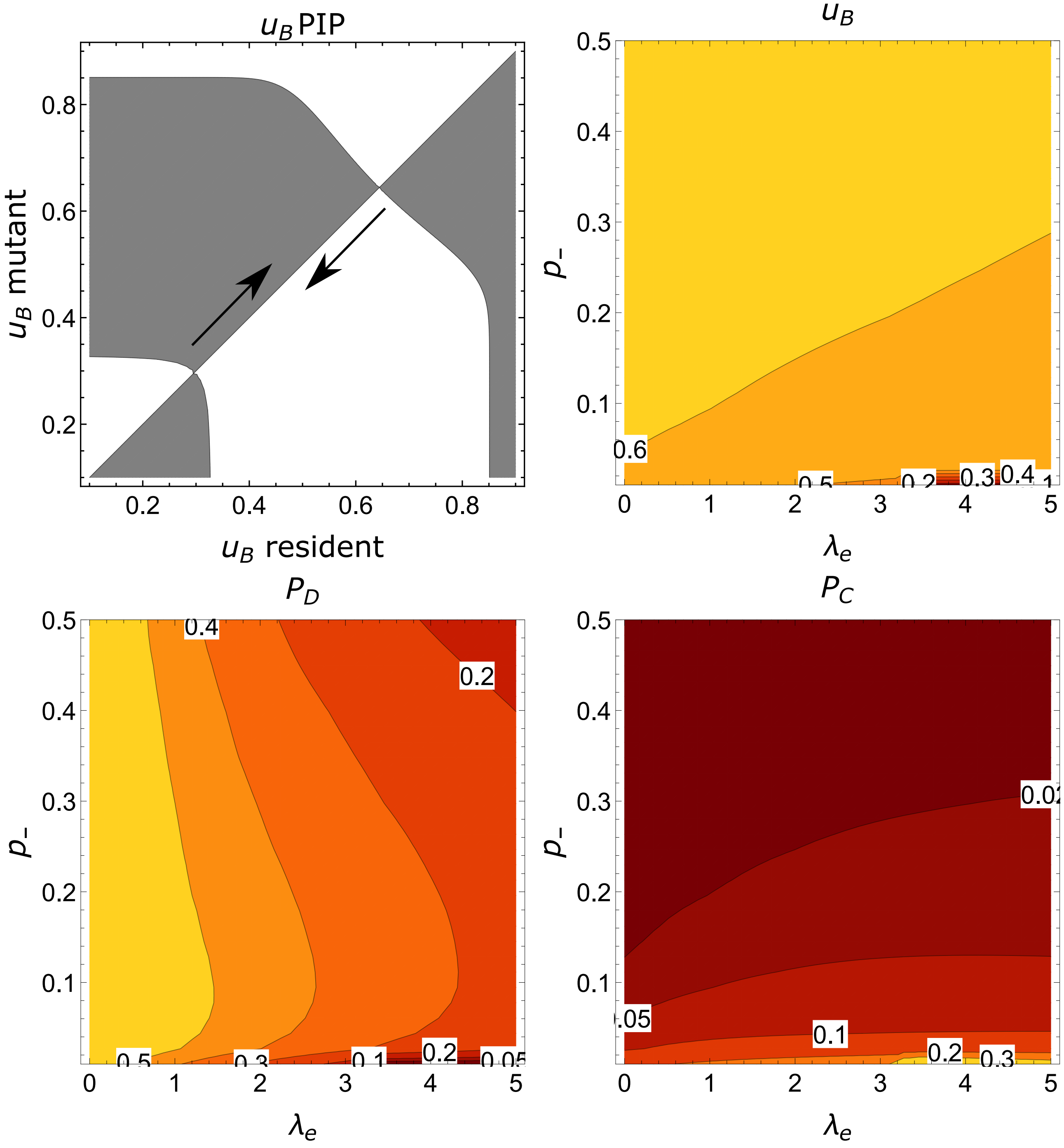

Figure S 5: Evolution of u_B. (a) Pairwise Invasibility Plot for the evolution of u_B, for the same model and parameter as in Fig.S2, with $u_D = 0.5$. The arrows indicate the direction of the singular points when λ_e increases or p_- decreases (here $p_- = 0.3, \lambda_e = 4$).(b-d) Contour plots for u_B^* (b), P_D (c) and P_C (d) for different values of λ_e (x-axis) and p_- (y-axis). Darker shades indicate smaller values, indicated by the labels reported in the plot. For all the figures we considered the same parameters as in Fig.S2, and $u_D^* = 0.5$.

Analogous conditions to the convergence and evolutionary stability can be formulated for the evolution of multiple traits. S^* is an ESS if and only if the Hessian H of the invasion fitness is negative definite. H is defined as:

$$H = \begin{pmatrix} H_{11} & H_{12} \\ H_{12} & H_{22}. \end{pmatrix} = \begin{pmatrix} \frac{\partial^2 \pi^S(u_D^{\bullet}, u_B^{\circ}, u_B^{\circ})}{\partial {u_D^{\bullet}}^2} & \frac{\partial^2 \pi^S(u_D^{\circ}, u_B^{\bullet}, u_B^{\circ})}{\partial u_B^{\bullet}\, \partial u_D^{\bullet}} \\ \frac{\partial^2 \pi^S(u_D^{\bullet}, u_B^{\bullet}, u_B^{\circ})}{\partial u_B^{\bullet}\, \partial u_D^{\bullet}} & \frac{\partial^2 \pi^S(u_D^{\circ}, u_B^{\bullet}, u_B^{\circ})}{\partial {u_B^{\bullet}}^2}. \end{pmatrix}_{u_D^{\bullet} = u_D^{\circ},\, u_B^{\bullet} = u_B^{\circ}} \tag{48}$$

For the evolution of multiple traits, convergence stability depends on the Jacobian of selection gradient J, in our case:

$$J = \begin{pmatrix} J_{11} & J_{12} \\ J_{21} & J_{22}. \end{pmatrix} = \begin{pmatrix} \frac{\partial D_{u_D}}{\partial u_D^{\circ}} & \frac{\partial D_{u_D}}{\partial u_B^{\circ}} \\ \frac{\partial D_{u_B}}{\partial u_D^{\circ}} & \frac{\partial D_{u_B}}{\partial u_B^{\circ}}. \end{pmatrix}_{u_B^{\circ} = u_B^*,\, u_D^{\circ} = u_D^*} \tag{49}$$

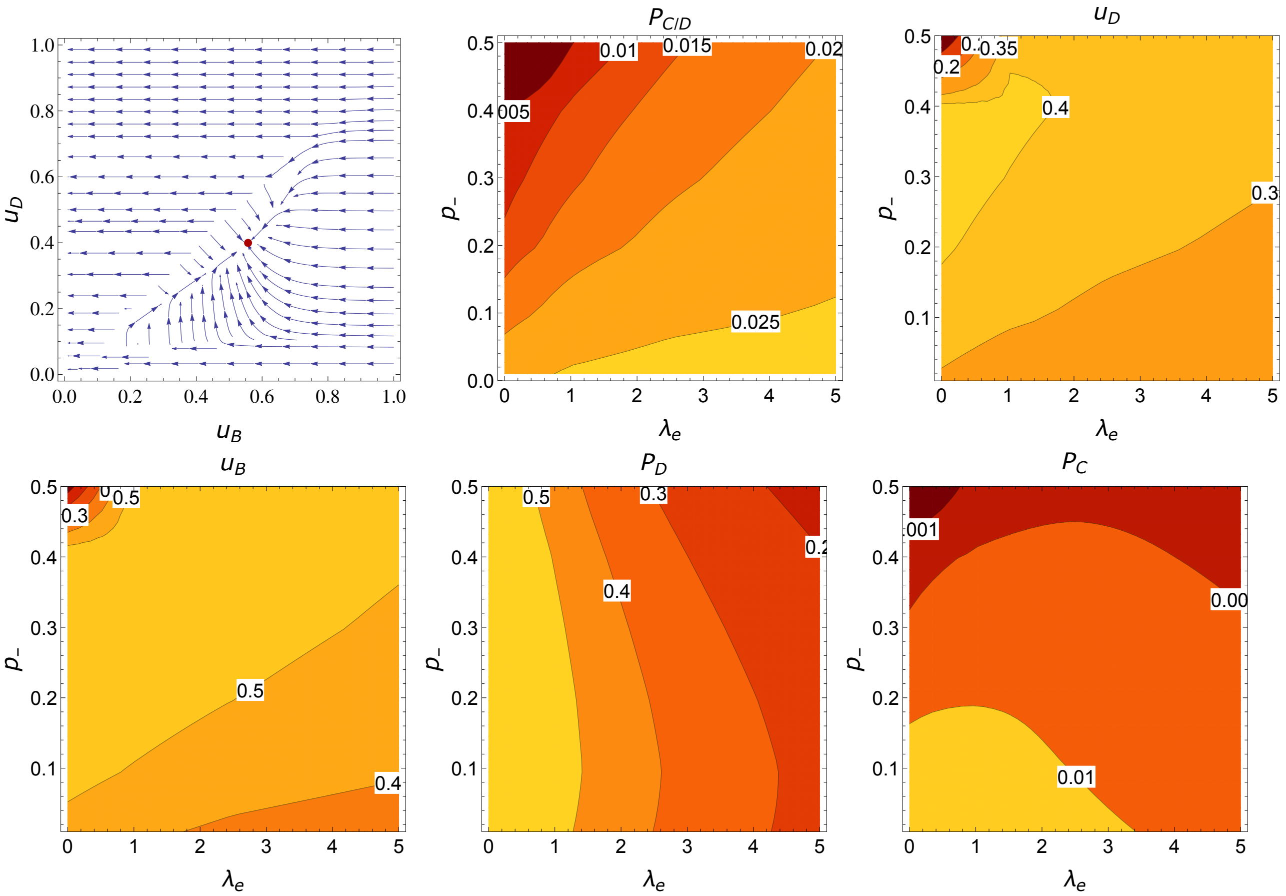

Figure S 6: Simultaneous evolution of u_D and u_B, for the same model and parameter as in Fig.S2. (a) Streamplot showing the evolutionary trajectories for equal trait variances and absent trait covariance, following the direction of the selection gradient. The red dot indicates the evolutionary attractor. Values of u_B and u_D are represented respectively on the x and y axes. (b-f) Countorplots for $\alpha(u_D^*, u_B^*)$ (b), u_D^* (c),u_B^* (d), P_D (e) and P_C (f) for different values of λ_e (x-axis) and p_- (y-axis). Darker shades indicate smaller values, indicated by the labels reported in the plot. For all the figures we considered the same parameters as in Fig.S2.

Strong convergence stability [15] guarantees convergence for the canonical deterministic adaptive dynamics, under the assumption that the mutational matrix varies gradually. This criterion is satisfied when J is negative definite. However it is possible to require a stronger criterion, guaranteeing that a singular point is robust to any gradualistic mutational path. When this criterion, defined as absolute convergence stability, is fullfilled, one does not need to take into account correlations between traits. Absolute convergence stability [16] is determined by the matrix $J' = J\,V$, where J is the Jacobian of the selection gradient and V is the mutational variance-covariance matrix:

$$V = \begin{pmatrix} V_{DD} & V_{DA} \\ V_{DA} & V_{AA} \end{pmatrix}. \tag{50}$$

When J' is negative definite, absolute convergence stability is satisfied. In order to ensure this, either V or J must be symmetric, if J is negative definite. However, absolute convergence stability is a very restrictive criterion, generally holding only for models with simple structure, since there are no reasons to expect J to be symmetric. Therefore, it is possible to adopt strong convergence stability and the ESS criterion to define a CSS in the multiple trait context[15].

Regarding the ESS condition, H is negative definite when its trace is negative and the determinant positive:

$$H_{11} + H_{22} < H_{12}^2, \tag{51}$$

$$H_{11}H_{22} > H_{12}^2. \tag{52}$$

Similarly for strong convergence stability, the Jacobian has to be negative definite. This is verified when:

$$\operatorname{tr} J = \left(\frac{\partial D_{u_D}}{\partial u_D^\circ} + \frac{\partial D_{u_B}}{\partial u_B^\circ} \right)_{u_B^\circ = u_B^* \,, u_D^\circ = u_D^*} < 0\,, \tag{53}$$

$$\det J = \left(\frac{\partial D_{u_D}}{\partial u_D^\circ}\frac{\partial D_{u_B}}{\partial u_B^\circ} - \frac{\partial D_{u_D}}{\partial u_B^\circ}\frac{\partial D_{u_B}}{\partial u_D^\circ} \right)_{u_B^\circ = u_B^* \,, u_D^\circ = u_D^*} > 0\,. \tag{54}$$

Due to the complex expression for the invasion fitness, it is not straightforward to obtain simple, readable stability conditions. However it is possible to draw some informal reasoning about the behavior of the system. First we note that with respect to the Hessian all terms but H_{22} can be simplified considering only the as-observer fitness π_{-}^{S}, since the as-actor component is independent of $u_D^\bullet$. The same reasoning can be applied to D_{u_D}, and hence to J_{11} and J_{12}. We also know that both H_{11} and J_{11} are always negative. We know as well that two possible singular points might exist. In general a singular point S^* exists, such that J_{22} and H_{22} are negative. For S^*, the traces of both H and J are negative. Therefore the singular point is either stable or a saddle. Given the tradeoffs

described for the single traits, a saddle is unlikely. This is also intuitively clear, by noticing that the mixed derivative terms are usually quite small: in our model an increase in u_D above u_D^* increases the proportion of C responses; however, a parallel increase in u_B buffers the risk C, partially inhibiting coordination. We verified this informal reasoning numerically. In Fig.S7 we show a representative part of the parameter space, showing that the largest eigenvalues for H and J are always negative, as long as environmental complexity is not so high that learning cannot occur anymore.

Evolution of u_D, u_B and temporal inhibition

Besides innate inhibitory mechanisms, organisms adjust their social behavior according to previous experience. As we have seen simulative strategies provide a useful tool to predict other's behavior when other sources of information are scarce, but as more social information is available, more direct strategies become advantageous. Empirically, several neurophysiological studies suggest that simulative circuits, relying on as-actor experience, and mechanisms relying on acquired as observers, are combined in mind-reading [17, 1]. For example, empirical studies have shown that empathy is strongly inhibited after interacting again with a defector stooge.

We investigate here the possibility that as-actor and as-observer informations are combined in a single mixed strategy. Specifically, we explore temporally mixed strategies, denoted as SP, initially adopting an S phenotype and later shifting to P, when enough social information learned as an observer

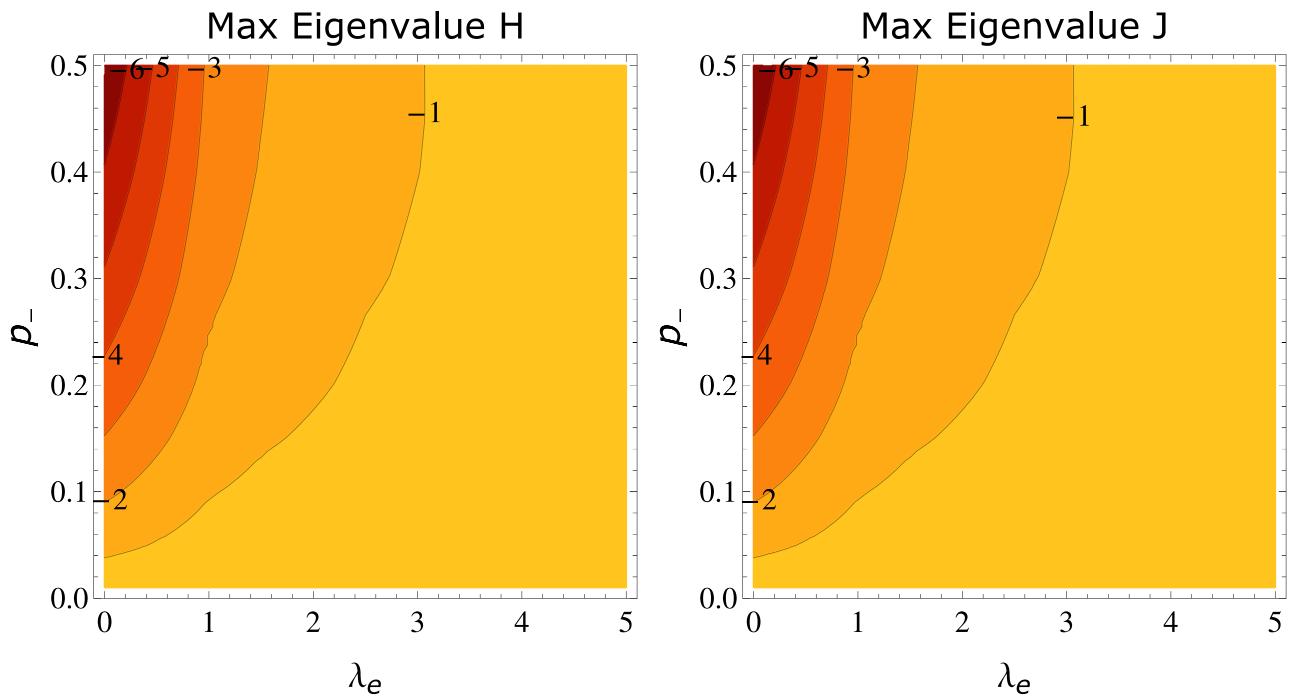

Figure S 7: Evolution of u_D for SP. Contour plots for the maximum eigenvalues for H (left) and J (right) for different values of λ_e (x-axis) and p_- (y-axis). Lighter regions indicate more simulative phenotypes.

becomes available. Such strategies employ a learned inhibitory mechanism, allowing individuals to stop using simulation and shift to a coordination-free strategy when the latter is sufficiently efficient. We model this by exploring the evolution of the trait u_S, defined as *shifting time*, the expected time at which a focal individual changes strategy type, from S to P: when $u_S \to 0$ the strategy converges to a pure P phenotype, while the higher the values of u_S the longer a simulative type is used before adopting a P phenotype.

We explore the evolution of such strategies, considering both evolvable traits u_D and u_B. We assume that an individual adopting SP adopts a P type whenever the payoff of adopting P is larger than by adopting S. Thus, for a focal individual, u_S depends on both u_B and u_D. u_S can be found by equating π^S and π^P:

$$E[p_B(x_+, t_+, u_B)\left(p_D^S(x_-, u_S, u_B^\bullet)d^- - p_C^S(u_D^\bullet x_-, u_S, u_B^\bullet)c^- - p_D^P(u_D^\bullet x_-, u_S, u_B^\bullet)d^-\right)] = 0. \quad (55)$$

The social fitness component of the switching type SP is simply given by the social fitness of S and P, respectively up to and after $t = u_S$:

$$\begin{aligned} \pi_-^{SP} &= \pi_-^S|_{t\leq u_S} + \pi_-^P|_{t>u_S} \\ &= p_-\, E[E_{t\leq u_S}[p_B^\circ\,(p_D^S\, d^- - p_C^S\, c^-)] + E_{t>u_S}[p_B^\circ\, p_D^P]\, d^-]. \end{aligned} \quad (56)$$

The selection gradient for SP is:

$$\frac{\partial \pi^{SP}}{\partial u_D^\bullet} = \frac{\partial \pi^S|_{t<u_S}}{\partial u_D^\bullet} \quad (57)$$

$$\frac{\partial \pi^{SP}}{\partial u_B^\bullet} = \frac{\partial \pi^S|_{t<u_S}}{\partial u_B^\bullet} + \frac{\partial \pi^P|_{t>u_S}}{\partial u_B^\bullet} \quad (58)$$

Therefore, the selection gradient for u_D is identical to what was analyzed so far, except that when u_S is 0 it evolves under random drift. For u_B, the as-actor component is also identical to the other strategies. The only difference is in the relative importance of the simulative strategy, now used only in the first part of an individual's lifetime. Therefore u_B evolves to slightly lower values than for a pure S strategy type (Fig.S9). Because of the similarity with the previous case, we perform again a sequential analysis of the evolvable traits , first by considering the evolution of a single trait, when the other is fixed, and later looking at the simultaneous evolution of both. However, we only show the quantitative differences, and focus on the different aspects between traits. Fig.S8 and Fig.S9 show respectively the PIP and a numerical exploration of the u_D-evolvable and u_B evolvable cases, presenting similar patterns to the ones observed for S types. However temporal inhibition allows us to investigate the dynamics of u_D and u_B without taking into account the frequencies of the different

strategy types. We can track the equilibrium values of u_S: the higher the value of u_S the longer an S phenotype is adopted by SP. Fig.S8a and Fig.S9b show how simulative phenotypes are extensively used over a wide range in parameter space. S phenotypes are always adopted, with the only exception of extreme values of p_- and λ_e. In fact when the probability of interactions is high and environmental variability are very low, P phenotypes are always more advantageous. At the opposite extreme, when p_- is low and λ_e very high, a focal individual is essentially a lone actor, almost never playing as an observer. Hence, u_B evolves towards minimum values, only optimizing the as-actor behavior.

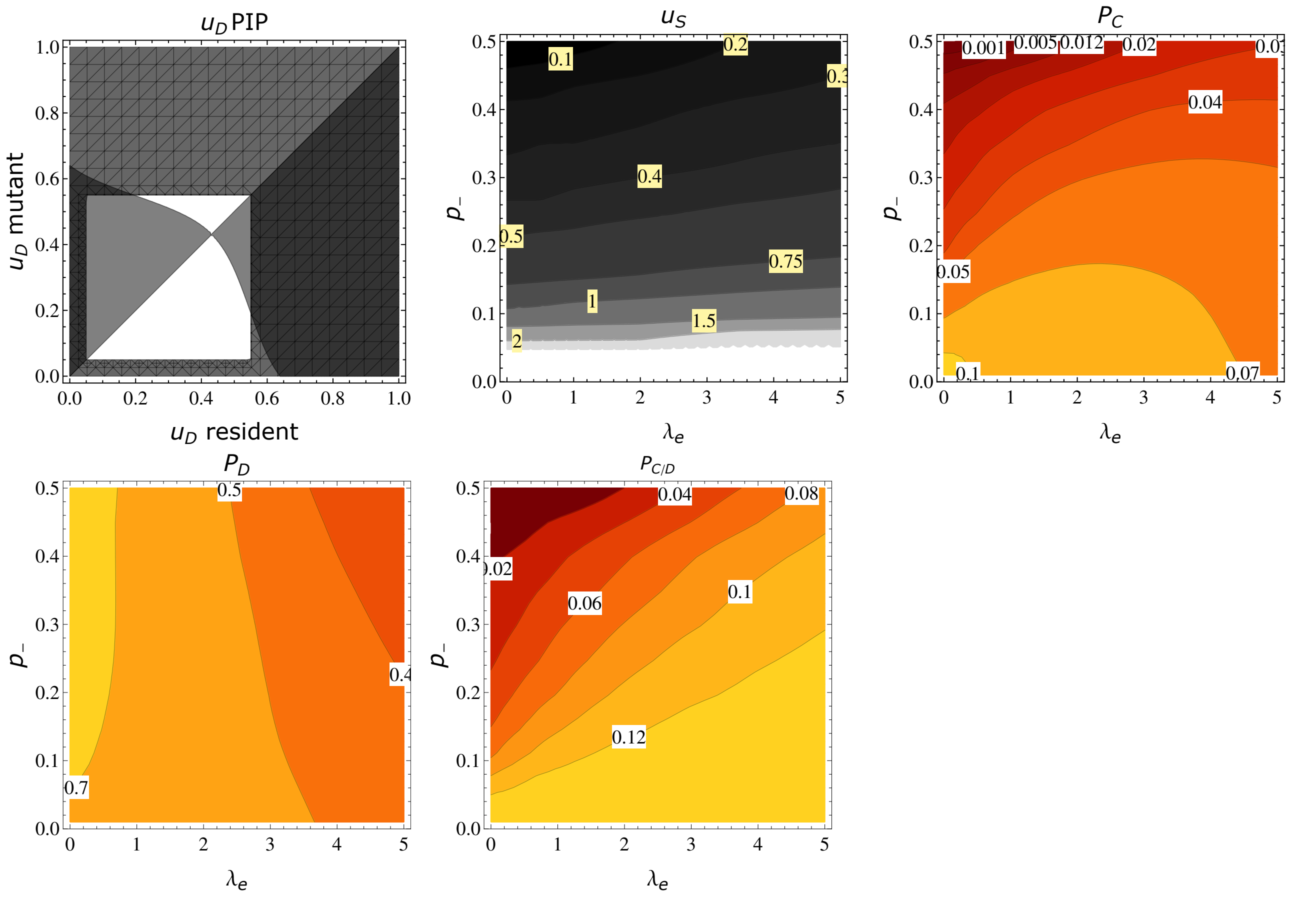

Figure S 8: Evolution of u_D for SP. (a) Pairwise Invasibility Plot (PIP) for the evolution of u_D. The values of u_D for the resident population and the mutant are reported respectively on the x and y axis. Gray regions indicate a higher fitness for the mutant, white ones for the resident population. Shaded areas indicate values of u_D for which either the mutant or the resident strategy never use a simulative phenotype. In those cases, u_D is only subjected to random drift. (b) Contour plots for u_S for different values of λ_e (x-axis) and p_- (y-axis). Lighter regions indicate more simulative phenotypes. (c-e) Contour plots for P_C (c), P_D (d) and P_{CD} for different values of λ_e (x-axis) and p_- (y-axis). Darker shades indicate smaller probability values, indicated by the labels reported in the plot. For all the figures we considered the same parameters as in Fig.S2.

We finally explore the case when u_D and u_B coevolve (Fig.S10). Also in this case, SP behaves similarly to S, regarding the evolution of u_D and u_B, for moderate values of p_- and λ_e. Despite the fact that inhibition could be achieved in multiple ways, small probabilities of coordination generally evolve. Interestingly, the presence of temporal inhibition determines a total inhibition of S when simulative

strategies become suboptimal. In this case, u_D evolves neutrally, while the selection gradient leads u_B to converge to minimal values. This leads to an evolutionary trap, and better combination of traits cannot evolve for simulative strategies unless mutations with big effects are possible. Therefore the system is bistable, since in this case an optimal combination of u_B and u_D for simulation cannot be achieved anymore.

The ratio between C and D responses increases for higher environmental variability and lower p_-, when simulative phenotypes are adopted for longer time. Interestingly the non-linear relation between p_- and λ_e and simulation is revealed in the equilibrium values of the traits u_D and u_B: although simulative strategies are used more frequently u_D^* and u_B^* decrease. This is due to the as-actor component of the fitness, becoming increasingly more important, and attempting to minimize the loss in B responses.

Regarding stability, the reasoning is analogous to the case of pure S strategies.

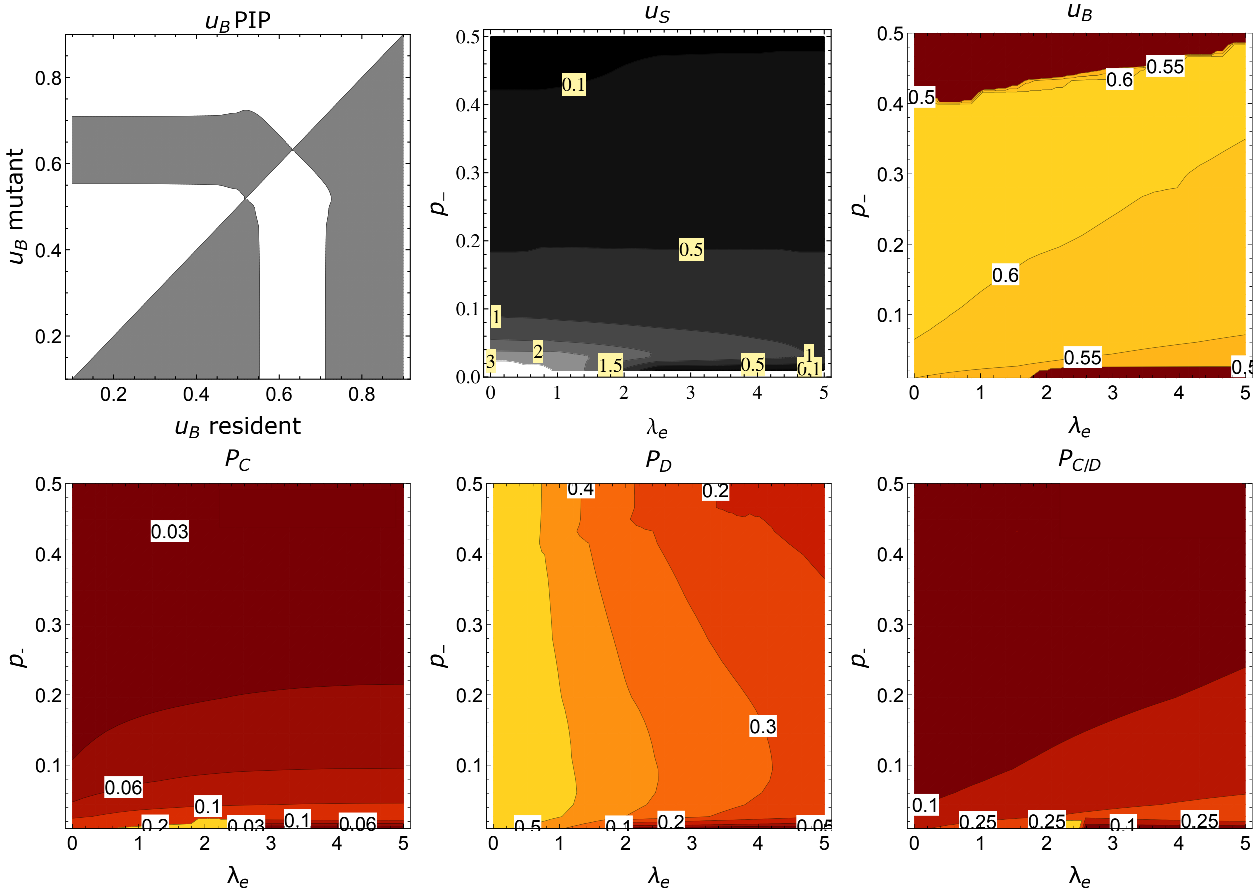

Figure S 9: Evolution of u_B for SP. (a) Pairwise Invasibility Plot (PIP) for the evolution of u_B. The values of u_D for the resident population and the mutant are reported respectively on the x and y axis. Gray regions indicate a higher fitness for the mutant, white ones for the resident population. (b) Contour plots for u_S for different values of λ_e (x-axis) and p_- (y-axis). Lighter regions indicate more simulative phenotypes. (c-f) Contour plots for u_B^* (c), P_C (d), P_D (e) and P_{CD} (f) for different values of λ_e (x-axis) and p_- (y-axis). Darker shades indicate smaller probability values, indicated by the labels reported in the plot. For all the figures we considered the same parameters as in Fig.S2 and $u_D = 0.5$.

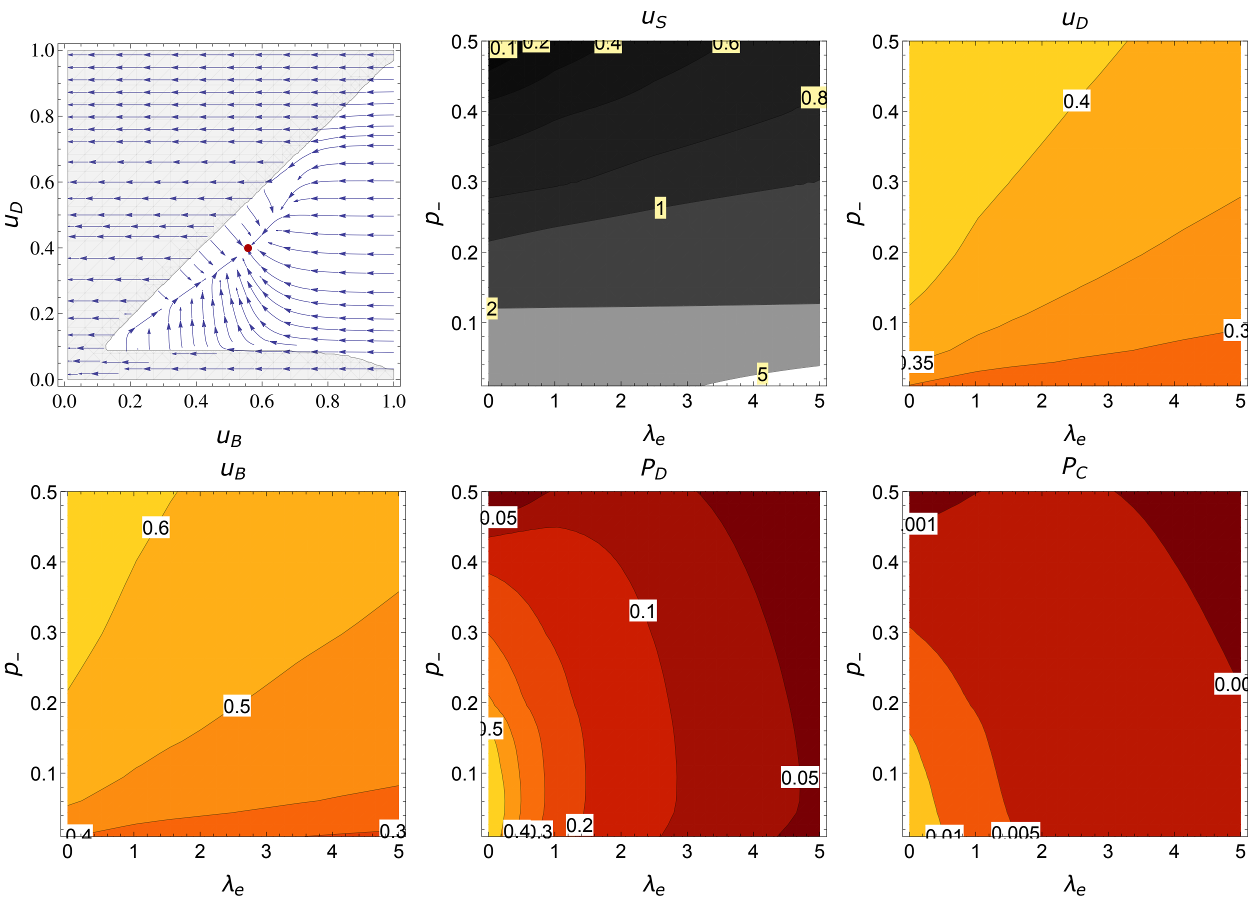

Figure S 10: Simultaneous evolution of u_D and u_B of SP, for the parameter as in Fig.S6. (a) Streamplot showing the evolutionary trajectories, following the direction of the selection gradient, assuming equal trait variances and absent trait covariance. The red dot indicates the evolutionary attractor. Values of u_B and u_D are represented respectively on the x and y axes. The shaded area indicates resident values of u_B and u_D for which simulative phenotypes are not used and u_D evolves neutrally. (b-f) Countorplots for $\alpha(u_D^*, u_B^*)$ (b), u_D^* (c),u_B^* (d), P_D (e) and P_C (f) for different values of λ_e (x-axis) and p_- (y-axis). Darker shades indicate smaller values, indicated by the labels reported in the plot. For all the figures we considered the same parameters as in Fig.S2.

Section 3

Kin selection and indirect benefits of coordination

With assortment, the social component of the fitness is obtained similarly to eq. 56:

$$\pi_- = \big(P_D\,(d^- - r\,d^+) + P_C\,(-c^- + r\,c^+) + r\,b\big)/(1+r) \quad . \tag{59}$$

Note that the optimal action D can vary depending on the probability of interacting with individuals with the same genotype, a property defined as assortment and captured by the assortment coefficient r. For example, a well known effect of assortment is to promote cooperation, that can become advantageous because of indirect benefits [18, 19]. Thus, it will be useful necessary to describe cases in which the appropriate response is to cooperate. Here we keep the notation used so far to describe the case without assortment, for which we assumed payoffs $+d^-, -d^+, -c^-, +c^+$ with all coefficients ≥ 0. Note however that the signs of the payoffs can be changed without loss of generality. Thus later, we will represent cases in which the appropriate response is to cooperate by defining the appropriate response as D_r, with payoffs $-d_r^-$ and d_r^+.

We first explore the evolution of full coordination and describe how the model is affected by different responses D and D_r, a case relevant when assortment is high and promotes cooperation; second, we explore the effects of r on the probability of coordination, for cases in which inhibition occurs.

Full coordination

C gives higher fitness than D when:

$$-c^- + r\,c^+ > \gamma_a(d^- - r\,d^+). \tag{60}$$

This can be written in a form that is reminiscent of conditions for the evolution of cooperation [18, 19]:

$$r > \frac{\gamma_a d^- + c^-}{\gamma_a d^+ + c^+}. \tag{61}$$

The numerator represents the costs of coordination for the observer: this depends both on the direct cost of coordination (c^-) and on the missed benefits from appropriate social responses ($\gamma_a d^-$). The denominator is given by the benefit for the recipient actor: this is the sum of the direct benefit of coordination (c^+) and the potential cost of being predicted and outcompeted socially by the observer ($\gamma_a d^+$). It can be useful also to think in terms of a cooperative response D_r, which has positive fitness

when $r > d_r^-/d_r^+$. In this case, C provides a higher fitness than D_r when:

$$\gamma_a < \frac{r\,c^+ - c^-}{r\,d_r^+ - d_r^-}\,. \tag{62}$$

Full coordination evolves more easily when γ_a is low, i.e. when a correct inference of the actor's state does not lead often to response D. This can occur when it is hard to learn the appropriate social response (section 4) or map the internal representation of an actor's action to an actual response. In these cases coordination provides a quick, although suboptimal, solution.

Effects of assortment on the probability of coordination

We investigate the effects of r on the recruitment of the as-actor network, u_D. For simplicity we neglect here the evolution of u_B, assuming it as a fixed trait. To estimate the effect of r on the evolutionary equilibrium for u_D^*, i.e. $\frac{du_D^*}{dr}$, we express the fitness gradient in terms of P_L, the expected probability of a correct inference about the actor's state i.e. $P_L^S = \Pr(\hat{a}_{+i}|s_{-i}) = E[p_B^\circ\, l^\bullet(u_D, t_+)] = P_C^S + P_D^S$. The fitness gradient is:

$$\frac{d\pi^S}{du_D} = \frac{dP_L^S}{du_D}\gamma_a\,(\tilde{d}^- - r\,\tilde{d}^+) - \frac{dP_C^S}{du_D}(\gamma_a\,\tilde{d}^- - c^- + r(-\gamma_a\,\tilde{d}^+ + c^+)), \tag{63}$$

We note again that coordination also implies an intrinsic cost, in term of missed D responses $(-\gamma_a\,d^- + r\,d^+)$. For the singular strategy, we can rearrange eq.63 into:

$$\frac{dP_C^S}{du_D}/\frac{dP_L^S}{du_D} = \gamma_a\,(\tilde{d}^- - r\,\tilde{d}^+)/(\gamma_a\,\tilde{d}^- - c^- + r(-\gamma_a\,\tilde{d}^+ + c^+)). \tag{64}$$

For $r = 0$ we recover eq.35, where the right hand term is just $\frac{d^-}{d^- + c^-}$. The derivative $\frac{du_D^*}{dr}$ can be calculated by implicit differentiation of the invasion fitness [3]. For clarity we express the invasion fitness as $F(u_D, r) = \frac{\partial \pi_-^S}{\partial u_D^\bullet}\big|_{u_D^\bullet = u_D}$, since in our model F does not depend on the resident trait. For a singular point, $F(u_D^*, r) = 0$. Hence:

$$\frac{\partial F(u_D^*, r)}{\partial u_D^*}\frac{du_D^*}{dr} + \frac{\partial F(u_D^*, r)}{\partial r} = 0, \tag{65}$$

Rearranging we obtain:

$$\frac{du_D^*}{dr} = -(\frac{\partial F(u_D^*, r)}{\partial r})/(\frac{\partial F(u_D^*, r)}{\partial u_D^*}) = 0. \tag{66}$$

Notice that $\frac{\partial F(u_D^*, r)}{\partial u_D^*} = \frac{\partial^2 \pi_-^S}{\partial^2 u_D^\bullet}\big|_{u_D^\bullet = u_D,\, u_D = u_D^*} < 0$ since u_D^* is a maximum of π_-^S and a CSS. Therefore in

this case:

$$\frac{du_D*}{dr} \propto \frac{\partial F(u_D^*, r)}{\partial u_D^*} = \frac{\partial}{\partial r}\left(\frac{\partial \pi_-^S(u_D^\bullet)}{\partial u_D^\bullet}\bigg|_{u_D^\bullet = u_D} \right)\bigg|_{u_D = u_D^*} \tag{67}$$

and $\frac{du_D^*}{dr} > 0$ holds when

$$(c^- + c^+ + (d^- + d^+)\gamma_a)\frac{dP_C^S}{du_D} - (d^- + d^+)\gamma_a > 0 \tag{68}$$

Substituting equation 64 for u_D^*, we find that u_D^* increase with r when

$$\frac{c^+ d^- - c^- d^+}{c^- + d^- \gamma_a - r(c^+ + d^+ \gamma_a)} > 0. \tag{69}$$

The denominator implies that a different effect of r occurs when eq.60 is satisfied, i.e. when r is high and C is the advantageous response. Since in this case an internal singular strategy does not exist we can neglect the denominator, considering only cases in which it is positive. Hence, eq.69 becomes simply $c^+ d^- - c^- d^+ > 0$. This condition can be easily interpreted in our standard case, when D is beneficial for the observer ($d^- > 0$) and detrimental for the actor ($d^+ < 0$):

$$\frac{d^-}{d^+} > \frac{c^-}{c^+}. \tag{70}$$

Increasing relatedness leads to an increase in coordination when the relative benefit of an appropriate social response (for the observer versus the actor) is larger than the relative cost of coordination. In nature this condition is likely fulfilled in most cases, since it is for the observer itself that a social response is more relevant, i.e. its payoff has the largest absolute value for the observer. In fact, in many cases a cost for the observed actor might not exist. When the appropriate social response is D_r, this condition is reversed and it reads as

$$\frac{d_r^-}{d_r^+} < \frac{c^-}{c^+}, \tag{71}$$

implying that increasing relatedness leads to an increase in coordination when a proper cooperative response provides a larger benefit to the actor than that determined by coordination. Therefore even when simulative strategies are employed for cooperation, as in empathy, an increase in coordination with r is observed. Note that the probability coordination, although to a minor extent, increases with assortment even if the best response is still defection. Remarkably, relatedness can even decrease the probability of coordination (Fig.S11). We also notice that at high u_D values, the sign of the fitness gradient is only determined by the C payoff: if C is rewarding ($r > c^-/c^+$), full coordination is evolutionarily stable. This condition is not mutually exclusive with the existence of an internal

singular strategy u_D^* (eq.60). Hence, the system is possibly bistable (Fig.4). As r or c^+/c^- increase the internal singular strategy increases its value, finally vanishing: in this case full coordination is the only evolutionarily stable strategy. The stability of full coordination when an internal singular strategy exist is due to the fact that when u_D is very high and beyond the activation threshold, small decreases in u_D do not reduce significantly the probability of coordination. Nevertheless, they might lead to a reduction in the probability of a correct inference about the actor's state. Clearly, this equilibrium is unstable for larger mutational steps, since C provides a lower fitness than the internal singular strategy.

Section 4

Simulation with as-observer learning

We investigated so far the case of simulative strategies employing only as-actor information, by assuming that an individual has a fixed probability of performing an appropriate social response given an accurate inference about the actor's state i.e. we treated the cognitive function γ_a as a fixed parameter. Here we extend our analyses to investigate the case in which a simulative observer learns

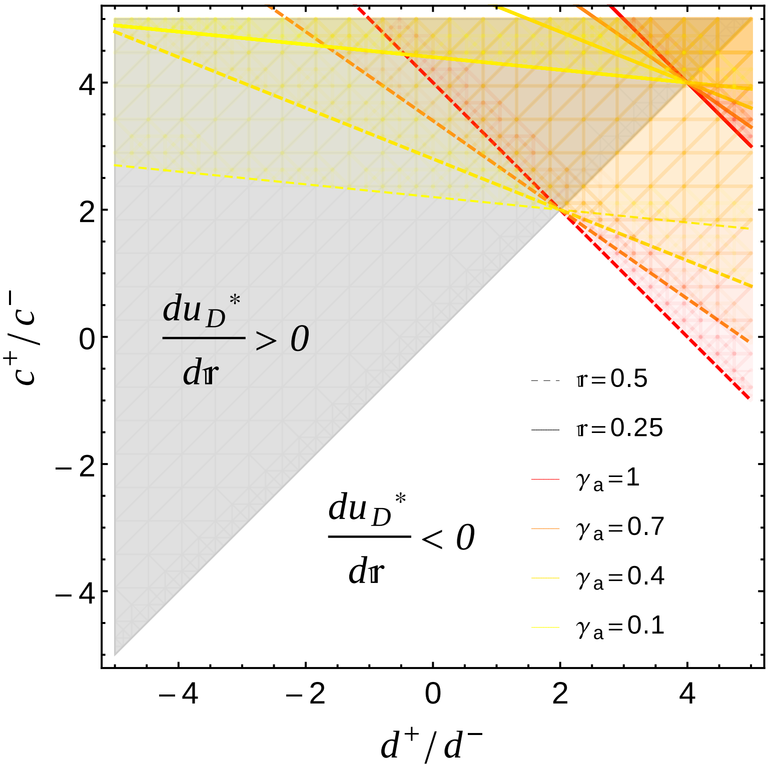

Figure S 11: Conditions for the empathic gradient ($\frac{du_D^*}{dr} > 0$, gray region) and for full coordination to evolve (yellow-red shaded regions) under different r (continous or dashed lines) and different γ_a (shades from yellow to red). In the gray regions, when r is low and C is not advantageous over $\tilde{D}$ (region not shaded in red-yellow), an increase in r leads to an increase in u_D^* and P_C. The opposite occurs in white unshaded regions. Above the colored lines indicating combinations or r and γ_a value, full coordination evolves and a singular strategy disappears.

in time what the appropriate social response is to an actor's action (a_{+i}). Hence, for simulative strategies, learning occurs in two phases: first an individual learns as an actor, and applies its as-actor network to infer an actor's action (a_{+i}) on the basis of social cue (s_{-i}) (Fig.1d, full gray arrow); secondly, when a correct inference is made ($\hat{a}_{+i}$), an observer learns at every social interaction what is the best social response (a_{-i}) (Fig.1d, second dashed arrow). Hence, the accuracy of social responses increase both with experience obtained as an actor (t_+) and as an observer (t_-). The former allows to map efficiently $\hat{s}_{-i}$ to $\hat{a}_{+i}$. The latter allows to infer the appropriate social response, $\hat{a}_{+i}$ to $\hat{a}_{-i}$.

In this model, the advantage of a correct inference about the actor's action is to facilitate the learning of the appropriate social response. This process can be seen as a reduction in the search space of possible social responses, since it is known what the actor's action is. Hence, we treat the cognitive function γ_a a learning function l, that to avoid confusion we denote as l_a. We reserve instead the symbol γ_a for a fixed parameter, describing the reduction of the search space for the appropriate social response, proportional to $(1-\gamma_a)$. The parameter γ_a has a similar effect to the constant case: when $\gamma_a \simeq 1$ knowing what the actor will do is perfectly informative about the appropriate social response, when $\gamma_a \simeq 0$ learning and social responses does not benefit of this information. The learning time for appropriate social responses, given a correct inference about the actor's state is then $t\,\tau_\gamma$, where:

$$\tau_\gamma = p_-\, E[p_B^\circ(x_+, t_+, \mathbf{u}) l^\bullet(u_D\, x_+, t_+)(1-\alpha^\bullet(u_D\, x_+, u_B))/(1-\gamma_a)] \tag{72}$$

We show here the behavior of this model, in the case where u_D evolves (Fig.S12). Simulative strategies still invade in highly variable environment, more easily the lower is p_-. However, since now the second step of social inferences depends on social learning, the range of p_- values for which they evolve is smaller. In particular, when γ_a decreases, fixed strategies can evolve even in highly variable environments because no learning either as-observer or as-actor learning limit the fitness of either strategies relying on learning, P and S (Fig.S12). When including relatedness, the same pattern of the constant γ_a is observed, with higher environmental complexity exerting the same effects of lower γ_a values, i.e. higher u_D evolve in response to relatedness.

Section 5

Agent Based Evolutionary Simulations

We tested the predictions of the deterministic model with an agent-based model, exploring the stochastic effects of a real learning algorithm and noise in perceived stimuli. We considered interactions, payoff structure and cognitive schemes analogous to the one presented along with the deterministic model

(Fig.1 and payoff Table 1; section 1 of the Appendix).

Agent structure

Individuals perceive stimuli as actors or observers (s_+ or s_-) with probability p_+ or p_-, respectively. We considered n different stimuli for actors, and a set of n corresponding stimuli for observers. In each turn each agent perceives a signal, represented as a vector of n stimuli intensities, one for each stimulus of the same class, either as-actor or as-observer. One of these stimuli is the leading one, determining the appropriate response. All the others are just noise. We assume the intensities of both leading and noise stimuli to be normally distributed, with mean μ_s and variance σ_s^2 for the formers, and μ_n and σ_n for the latters. For the simulations shown in the following figures, we assumed $\mu_s = 0.5, \sigma_s = 0.1, \mu_n = \mu_s/10, \sigma_n = \sigma_s/10$.

Individuals select a response to a given stimulus representation, using a $n \times n$ association matrix M of weights q_{ij}. Specifically, each element q_{ij} is a weight determining the strength of the association between a stimulus representation $\hat{s} = i$ and action representation $\hat{a} = j$. Therefore, after a signal is perceived, an action representation is selected probabilistically according to a softmax action selection function l, that take into account the intensity of the signal and the weights associated to the corresponding actions. In particular, action j is chosen with probability

$$Pr(\hat{a} = j | x_1\, q_{1j}, \ldots, x_i\, q_{ij}, \ldots, x_n\, q_{nj}) = \frac{e^{\sum_i^n x_i\, q_{ij}/T}}{\sum_{j=1}^n e^{\sum_i^n x_i\, q_{ij}/T}} \tag{73}$$

where the weights of a response to a given stimulus i are weighted for the intensity of the relative signal x_i. T defines the temperature of the softmax function. As T increases, the more exploratory is the

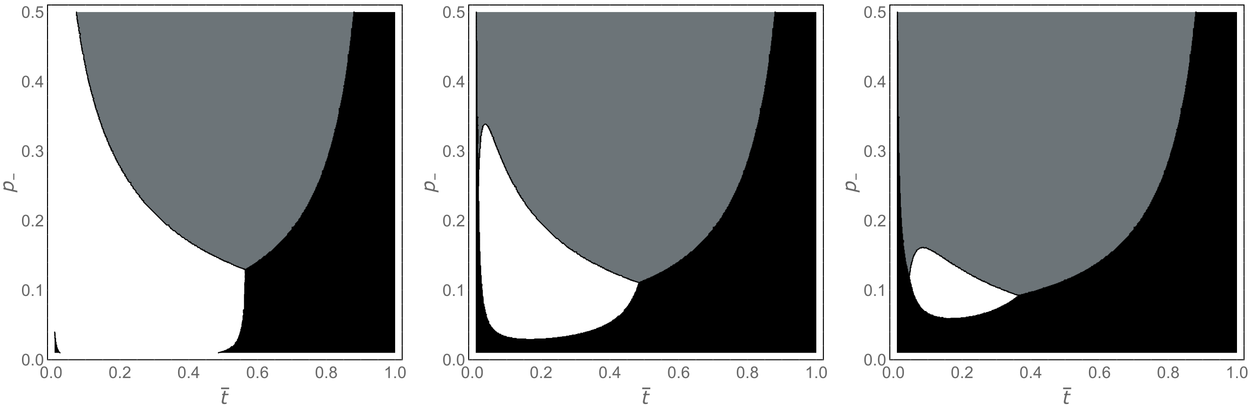

Figure S 12: **Competition among strategies in the simulation with as-observer learning case.** Invasion domains for S (white), P (grey) and F (black) for different values of γ_a. From left to right, γ_a equals 0.98, 0.9 and 0.8. On the y-axis p_- varies, while on the x-axis $\bar{t}$ changes as a function of λ_e ($\bar{t} = 1/(\lambda_d + \lambda_e)$). We considered the same model as in Fig.1, with sigmoid activation function and a learning curve as in Table 2. All the other parameters are the same as in Fig.S2.

action selection process, selecting occasionally responses with lower weights, and allowing the algorithm to be flexible and robust to initial errors. When T decreases, the algorithm is more conservative, choosing almost always the action with the highest weight.

Weights are updated accordingly to previous experience and payoffs, so that an agent's responses converge to the appropriate ones in time as learning occurs. When an individual performs an appropriate response to the perceived stimulus, it updates the corresponding weights with the learning rule

$$q_{i,j}(t+1) = (1-\alpha_i)q_{i,j}(t) + \alpha_i\, r, \tag{74}$$

where $\alpha_i = \alpha\,\frac{x_i}{\sum_j^n x_j}$ and α is a constant. The reward r at time t is 1 if the response performed is correct (thus providing a positive payoff), and $r = 0$ if incorrect. Thus, when an agent perceives a stimulus $s = \pm i$, it chooses probabilistically a response on the base of its previous history of rewards. All the as-actor stimuli $s \in S^+$,e.g. an as-actor stimulus s_{+i}, are associated to different possible actions $a \in A^+$, e.g. a_{+j}, according to the weights of a non-social matrix M^+ ($M^+|\quad \hat{S}^+ \to \hat{A}^+$). Social stimuli $s \in S^-$ are instead processed differently by the different mind-reading strategies, organized as Fig.1 analogously to the deterministic model. All strategies share the same learning rule, and the only differences are due to the organization of the mind-reading circuits:

- An F-strategy associates social stimuli to the their responses by means of a social matrix M^- ($M^-|\quad \hat{S}^- \to \hat{A}^-$). The weights are fixed within a generation, hence not learned but evolved.
- A P-strategy associates social stimuli to their responses by means of a social matrix M^- ($M^-|\quad \hat{S}^- \to \hat{A}^-$). The weights reflect past social experience, being updated after each social interaction.
- An S-strategy takes advantage of the as-actor experience, in the form of matrix M^+, in order to interpret social stimuli. Therefore, it first maps a social stimulus representation to a non-social stimulus representation by rescaling the stimulus intensity by an evolvable scalar factor u_D. The simulated as-actor stimulus, taking advantage of M^+, is mapped to an as-actor representation.

An activation function α is implemented analogously to the deterministic case. In this case we consider a step function with an evolvable activation threshold u_B. We notice that analogously to the deterministic case, P and S strategies differ as they use as-observer or as-actor experience, i.e. M^- and M^+, respectively. Regarding F strategies we assumed, conservatively against the invasion of S and P, that they are perfectly adapted to one of the experienced environmental states, by fixing the weights of the M^-. For small n we let the single weights to evolve independently, obtaining comparable results.

Each individual experiences 500 stimuli, distributed across a number n_e of identically distributed environmental states.

Selection scheme

Each simulation occurs in two stages. In a first stage the strategies interact and compete within the same strategy type for 10 generations. In this phase the traits u_D and u_B evolve to an average value for each strategy, since u_D and u_B can mutate (independent mutation rates $\mu(u_D) = \mu(u_\alpha) = 0.2$). When a mutation occurs the current value of the trait is added a random number between -0.2 and 0.2. The strategy types are mutually exclusive, so that an individual is either S, F or P. In a second stage, 2000 agents for each strategy start interacting and competing for 25 generations. Selection occurs through a softmax function with temperature $T_s = 0.1$, weighing individuals'total payoff.

Results

Evolutionary simulations with populations characterized by different strategic types, i.e. F, P and S, and continuous traits u_D and u_B traits, are performed, and the frequencies of the different strategies and traits are tracked over time. The results of the deterministic model are supported, with S-strategies dominating in more complex environments (bottom row, Fig.S13), where social information might be insufficient for accurate inferences. Contrarily, F-strategies dominate in very simple environments (top row), and P-strategies evolve for intermediate levels of environmental variability and higher effective interaction rates (central row, central and right columns). Confirming the predictions of the deterministic model, the trait values evolve to intermediate levels only in S-strategies (Fig.S14). Vice versa, u_B converges to minimal value in F and P strategies. u_D is expressed only in simulative strategies, therefore it evolves neutrally in F and P. When S dominates small levels of coordination are sustained (Fig.S15). For the parameters here explored, agents coordinate about 1% of the social interactions.

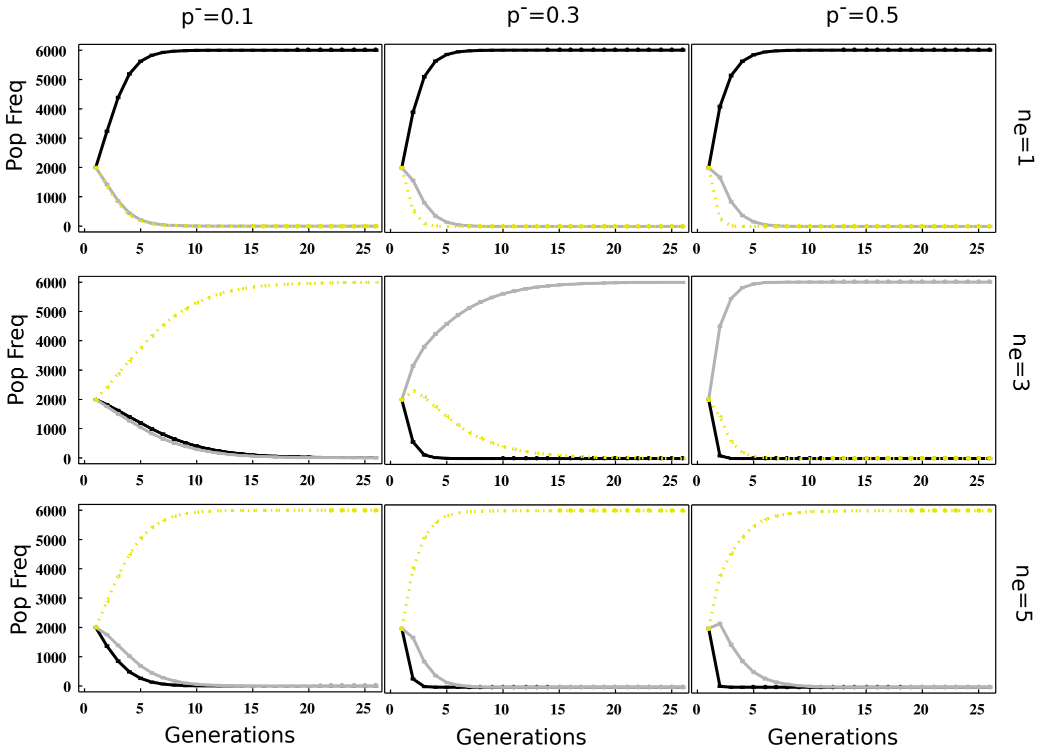

Figure S 13: Evolutionary simulations. Number of individuals adopting F (black), P (grey) and S (dotted yellow) strategies (y-axis) over time, measured in generations (x-axis). Rows indicates different numbers of environmental states visited by the individuals (environmental variability), columns different values of p_{-}, the probability of an informative social interaction. The parameters used for the simulations are $d = 5, c = 2, T = 0.05, \alpha = 0.2$.

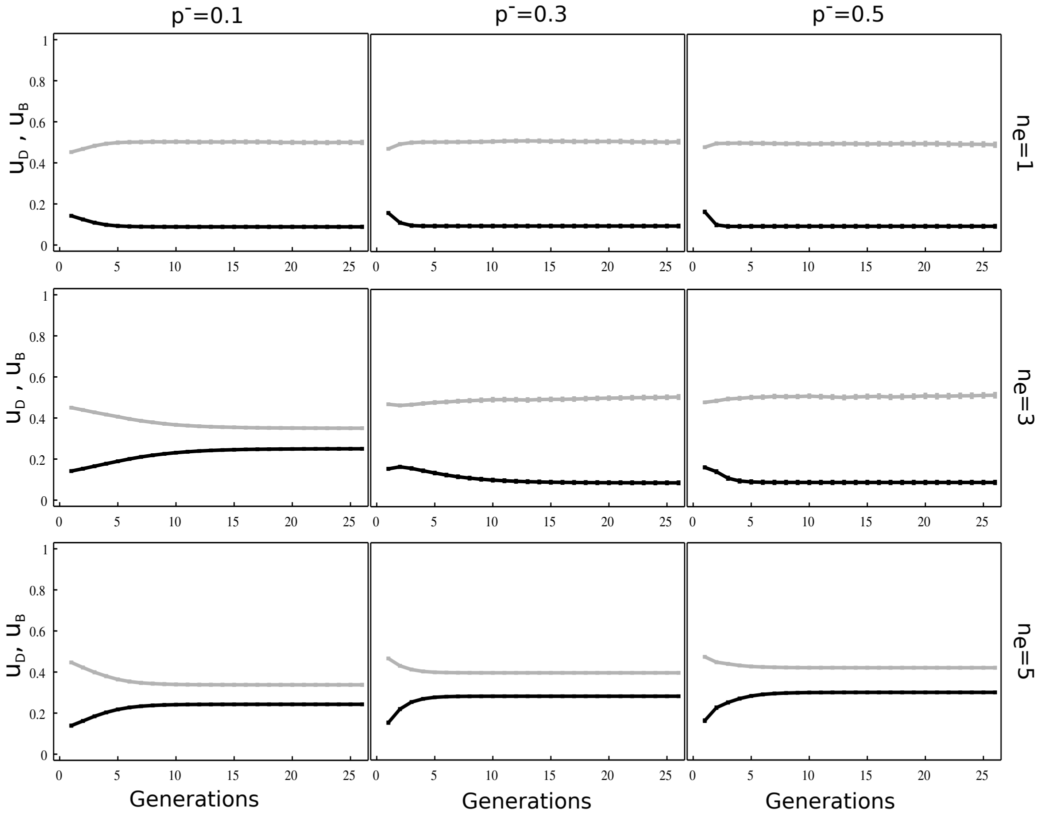

Figure S 14: Evolution of the traits u_D (gray) and u_B (black) for the evolutionary simulations shown in Fig.S13.

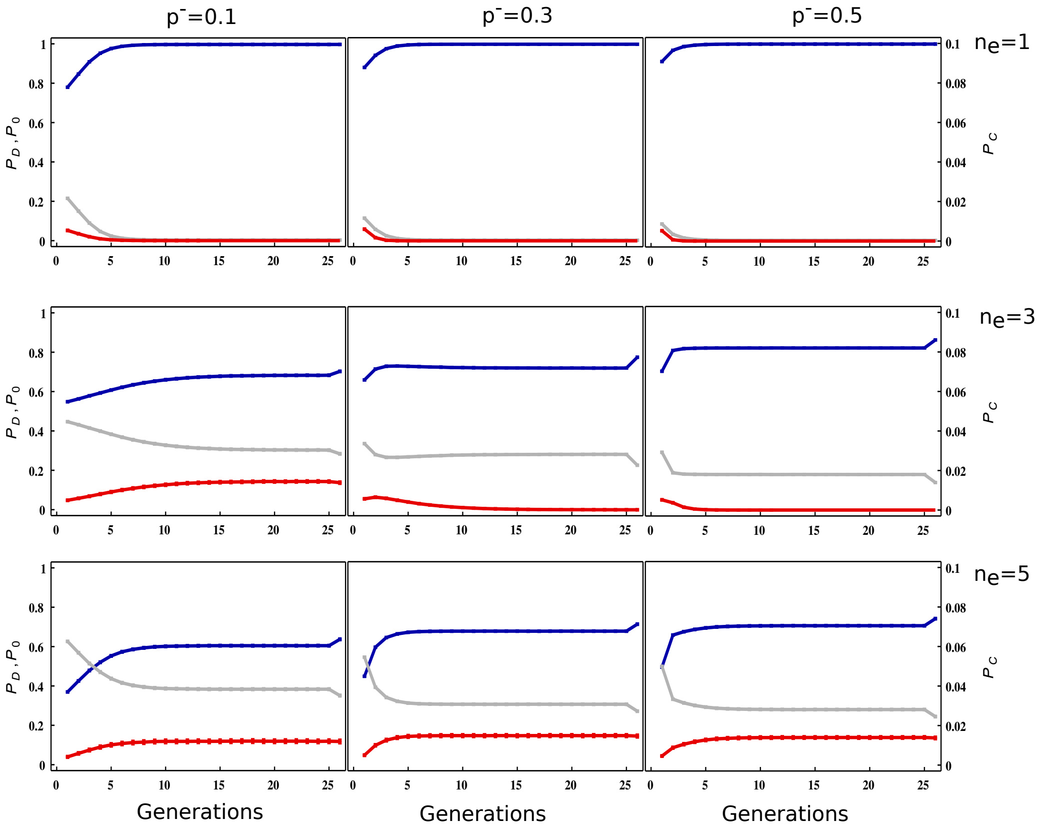

Figure S 15: Average defection (P_D, blue), inappropriate response (P_0, gray) and cooperation (P_C, red) over generations.

References

[1] H. G. Engen and T. Singer, "Empathy circuits," Current Opinion in Neurobiology, vol. 23, pp. 275–282, Apr. 2013.

[2] S. Geritz, J. Metz, . Kisdi, and G. Meszéna, "Dynamics of Adaptation and Evolutionary Branching," Physical Review Letters, vol. 78, pp. 2024–2027, Mar. 1997.

[3] S. P. Otto and T. Day, A Biologist's Guide to Mathematical Modeling in Ecology and Evolution. Princeton University Press, 2007.

[4] O. Diekmann, "A beginners guide to adaptive dynamics," Summer School on Mathematical Biology, pp. 63–100, 2002.

[5] D. E. Rumelhart, G. E. Hinton, and R. J. Williams, "Learning representations by back-propagating errors," Nature, vol. 323, pp. 533–536, Oct. 1986.

[6] V. Kůrková, "Kolmogorov's theorem and multilayer neural networks," Neural Networks, vol. 5, pp. 501–506, Jan. 1992.

[7] J. Han and C. Moraga, "The influence of the sigmoid function parameters on the speed of back-propagation learning," in From Natural to Artificial Neural Computation (J. Mira and F. Sandoval, eds.), Lecture Notes in Computer Science, pp. 195–201, Springer Berlin Heidelberg, 1995.

[8] B. Karlik and A. Vehbi, "Performance Analysis of Various Activation Functions in Generalized MLP Architectures of Neural Networks," International Journal of Artificial Intelligence and Expert Systems (IJAE), vol. 1, no. 4, pp. 111–122, 2011.

[9] A. Serwa, "Studying the Effect of Activation Function on Classification Accuracy Using Deep Artificial Neural Networks," Journal of Remote Sensing & GIS, vol. 06, no. 03, 2017.

[10] L. Zhang, "Implementation of Fixed-point Neuron Models with Threshold, Ramp and Sigmoid Activation Functions," IOP Conference Series: Materials Science and Engineering, vol. 224, no. 1, p. 012054, 2017.

[11] R. Rojas, Neural Networks: A Systematic Introduction. Berlin Heidelberg: Springer-Verlag, 1996.

[12] S. Haykin and S. S. Haykin, Neural Networks and Learning Machines. Prentice Hall, 2009. Google-Books-ID: K7P36lKzI_QC.

[13] M. A. Nielsen, Neural Networks and Deep Learning. 2015.

[14] F. Chersi, P. F. Ferrari, and L. Fogassi, “Neuronal Chains for Actions in the Parietal Lobe: A Computational Model,” PLoS ONE, vol. 6, p. e27652, Nov. 2011.

[15] O. Leimar, “Multidimensional convergence stability,” Evolutionary Ecology Research, vol. 11, pp. 191–208, 2009. Questions: Are there general stability conditions for the evolution Of Multidimensional traits, regardless of genetic correlations between traits? Can genetic correlations influence whether evoluti ...

[16] V. Ravigné, U. Dieckmann, and I. Olivieri, “Live Where You Thrive: Joint Evolution of Habitat Choice and Local Adaptation Facilitates Specialization and Promotes Diversity.,” The American Naturalist, vol. 174, pp. E141–E169, Oct. 2009.

[17] C. Lamm, J. Decety, and T. Singer, “Meta-analytic evidence for common and distinct neural networks associated with directly experienced pain and empathy for pain,” NeuroImage, vol. 54, pp. 2492–2502, Feb. 2011.

[18] M. A. Nowak, “Five rules for the evolution of cooperation,” Science (New York, N.y.), vol. 314, pp. 1560–1563, Dec. 2006.

[19] C. Taylor and M. A. Nowak, “Transforming the dilemma,” Evolution, vol. 61, pp. 2281–2292, Oct. 2007.
